# Correction: Gestational age at birth and body size from infancy through adolescence: An individual participant data meta-analysis on 253,810 singletons in 16 birth cohort studies

**DOI:** 10.1371/journal.pmed.1004232

**Published:** 2023-04-18

**Authors:** Johan L. Vinther, Tim Cadman, Demetris Avraam, Claus T. Ekstrøm, Thorkild I. A. Sørensen, Ahmed Elhakeem, Ana C. Santos, Angela Pinot de Moira, Barbara Heude, Carmen Iñiguez, Costanza Pizzi, Elinor Simons, Ellis Voerman, Eva Corpeleijn, Faryal Zariouh, Gilian Santorelli, Hazel M. Inskip, Henrique Barros, Jennie Carson, Jennifer R. Harris, Johanna L. Nader, Justiina Ronkainen, Katrine Strandberg-Larsen, Loreto Santa-Marina, Lucinda Calas, Luise Cederkvist, Maja Popovic, Marie-Aline Charles, Marieke Welten, Martine Vrijheid, Meghan Azad, Padmaja Subbarao, Paul Burton, Puishkumar J. Mandhane, Rae-Chi Huang, Rebecca C. Wilson, Sido Haakma, Sílvia Fernández-Barrés, Stuart Turvey, Susana Santos, Suzanne C. Tough, Sylvain Sebert, Theo J. Moraes, Theodosia Salika, Vincent W. V. Jaddoe, Deborah A. Lawlor, Anne-Marie Nybo Andersen

The fifth author’s name is indexed incorrectly. The author’s name should be indexed as Sørensen TIA. The correct citation is: Vinther JL, Cadman T, Avraam D, Ekstrøm CT, Sørensen TIA, Elhakeem A, et al. (2023) Gestational age at birth and body size from infancy through adolescence: An individual participant data meta-analysis on 253,810 singletons in 16 birth cohort studies. PLoS Med 20(1): e1004036. https://doi.org/10.1371/journal.pmed.1004036
