# Supplementary figures and images for: Gestational age at birth and body size from infancy through adolescence: An individual participant data meta-analysis on 253,810 singletons in 16 birth cohort studies
Source: PLoS Med. 2023 Jan 26;20(1):e1004036. doi: 10.1371/journal.pmed.1004036 (PMC9879424; doi:10.1371/journal.pmed.1004036)

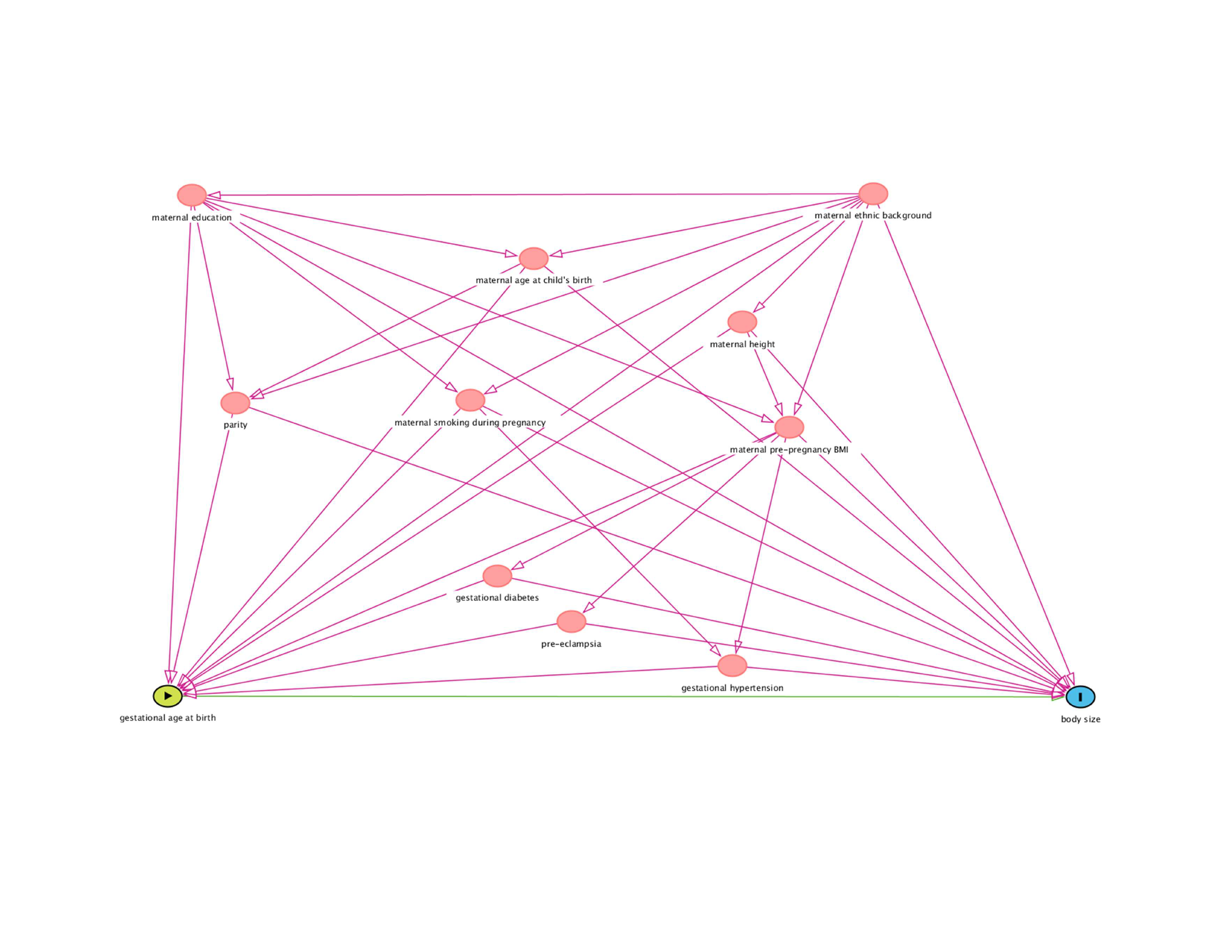

Supplement: S1 Fig — (TIF) [file pmed.1004036.s001.tif]

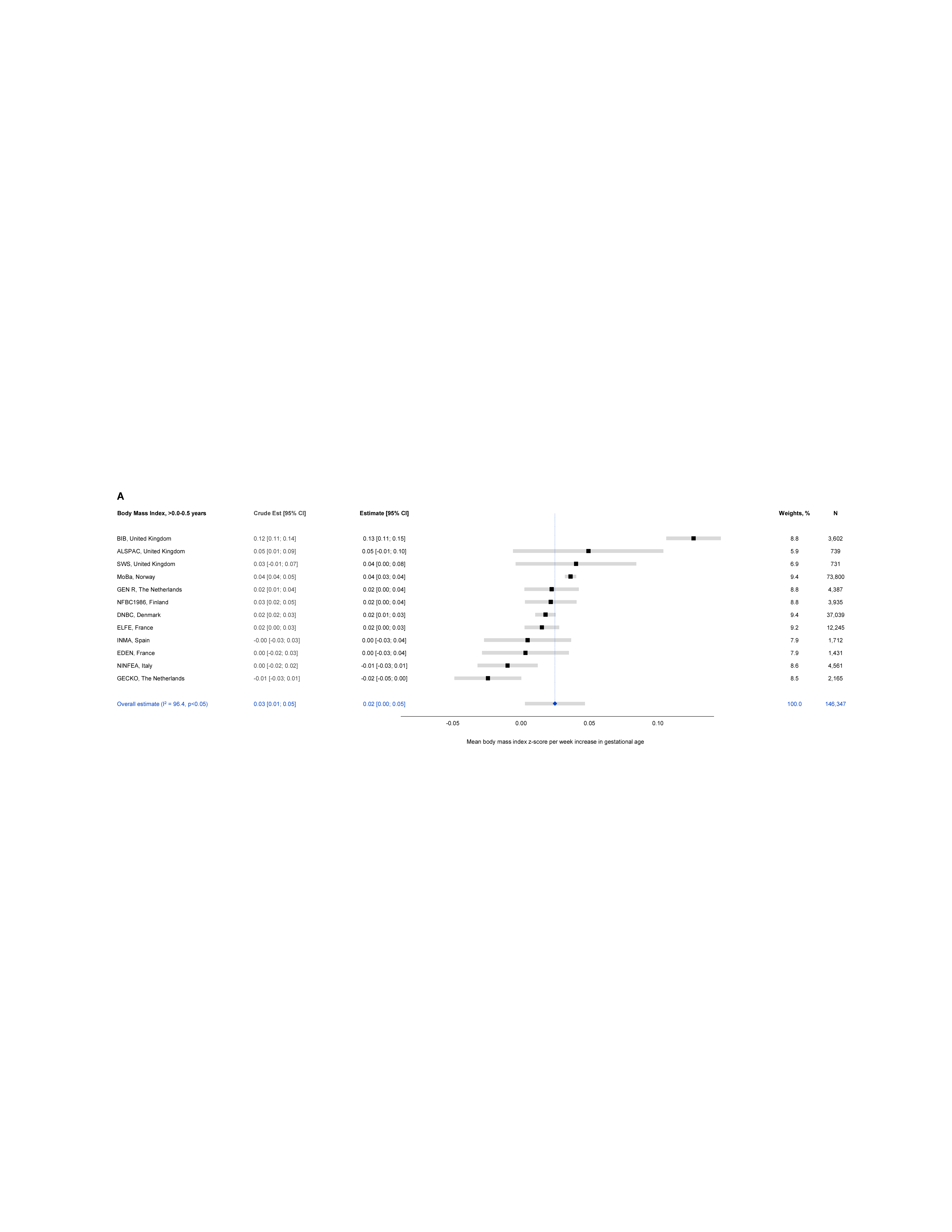

Supplement: S2 Fig — Unadjusted and adjusted estimates with 95% CIs from study-specific linear regression models, where cohorts were assigned weights under the random-effects model to attain the overall estimates. The dot in the forest plot represents the adjusted estimates, while the whiskers span the 95% CI, whereas I2-statistics (I2), sample size (N), studies, and p-value (two-sided, <0.05) relate to adjusted estimates. Estimates reflect mean differences in BMI z-score per week increase in GA at birth in (A) early infancy (>0.0–0.5 years), (B) late infancy (>0.5–2.0 years), (C) early childhood (>2.0–5.0 years), (D) mid-childhood (>5.0–9.0 years), (E) late childhood (>9.0–14.0 years), and (F) adolescence (>14.0–19.0 years). Models are adjusted for sex of child, and the following maternal characteristics: age at child’s birth, education, height, prepregnancy BMI, smoking during pregnancy, parity, ethnic background, gestational diabetes and hypertension, and preeclampsia. Cohort-specific estimates were adjusted for the maximum available set of the confounding variables (see Table 1). BMI, body mass index; CI, confidence interval; GA, gestational age. (TIF) [file pmed.1004036.s002.tif]

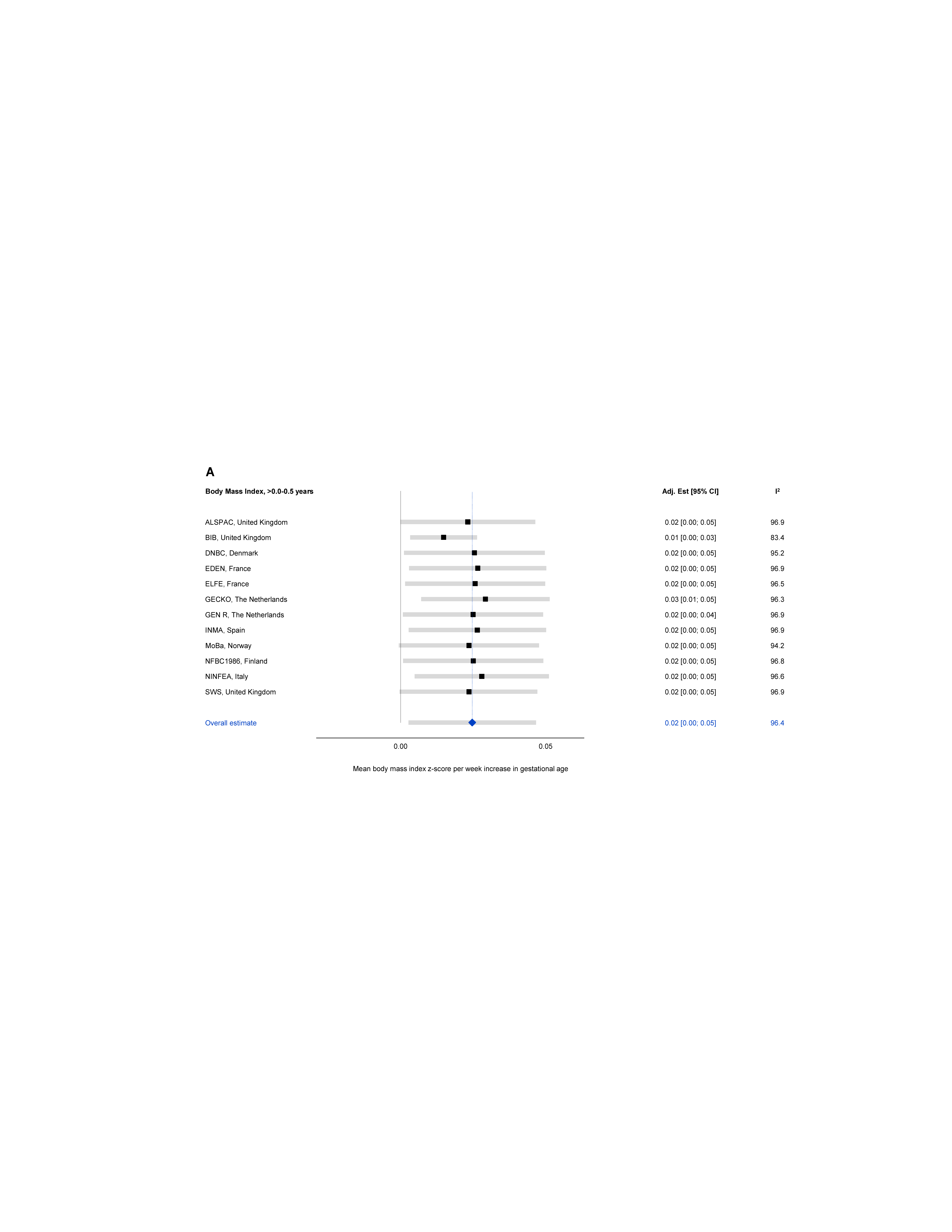

Supplement: S3 Fig — Overall adjusted estimates of BMI z-score with 95% CIs from study-specific linear regression models, where cohorts were assigned weights under the random-effects model to attain the estimate. The dot in the forest plot represents the adjusted estimates, while the whiskers span the 95% CI, whereas I2-statistics (I2), sample size (N), studies, and p-value (two-sided, <0.05) relate to adjusted estimates. Estimates reflect mean differences in BMI z-score per week increase in GA at birth in (A) early infancy (>0.0–0.5 years), (B) late infancy (>0.5–2.0 years), (C) early childhood (>2.0–5.0 years), (D) mid-childhood (>5.0–9.0 years), (E) late childhood (>9.0–14.0 years), and (F) adolescence (>14.0–19.0 years). Models are adjusted for sex of child, and the following maternal characteristics: age at child’s birth, education, height, prepregnancy BMI, smoking during pregnancy, parity, ethnic background, gestational diabetes and hypertension, and preeclampsia. Cohort-specific estimates were adjusted for the maximum available set of the confounding variables (see Table 1). BMI, body mass index; CI, confidence interval; GA, gestational age. (TIF) [file pmed.1004036.s003.tif]

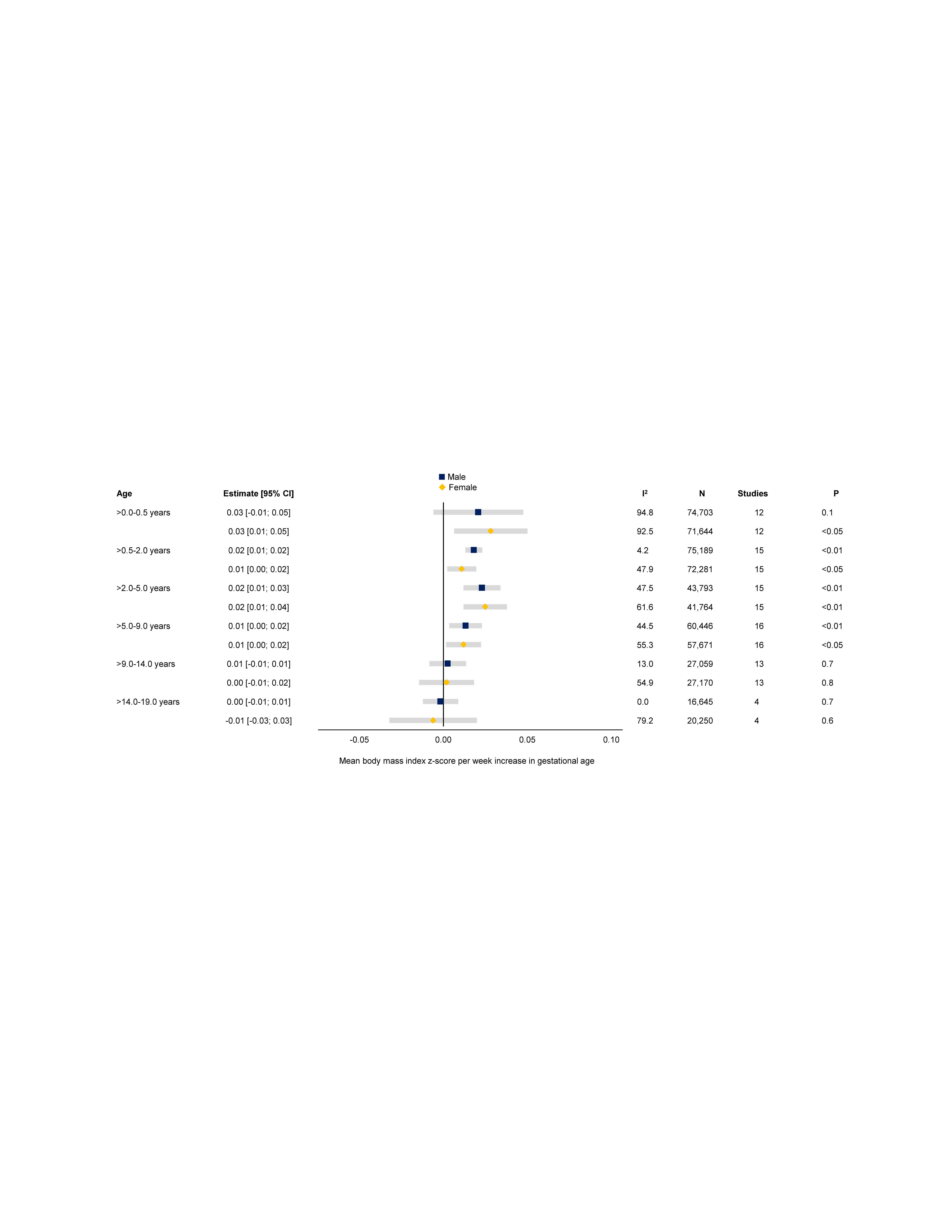

Supplement: S4 Fig — Overall adjusted estimates of BMI z-score with 95% CIs from study-specific linear regression models, where cohorts were assigned weights under the random-effects model to attain the estimate. The blue (male) and yellow (female) dots in the forest plot represent the adjusted estimates, while the whiskers span the 95% CI, whereas I2-statistics (I2), sample size (N), studies, and p-value (two-sided, <0.05) relate to adjusted estimates. Estimates reflect mean differences in BMI z-score per week increase in GA at birth in early infancy (>0.0–0.5 years), late infancy (>0.5–2.0 years), early childhood (>2.0–5.0 years), mid-childhood (>5.0–9.0 years), late childhood (>9.0–14.0 years), and adolescence (>14.0–19.0 years). Models are adjusted for sex of child, and the following maternal characteristics: age at child’s birth, education, height, prepregnancy BMI, smoking during pregnancy, parity, ethnic background, gestational diabetes and hypertension, and preeclampsia. Cohort-specific estimates were adjusted for the maximum available set of the confounding variables (see Table 1). BMI, body mass index; CI, confidence interval; GA, gestational age. (TIF) [file pmed.1004036.s004.tif]

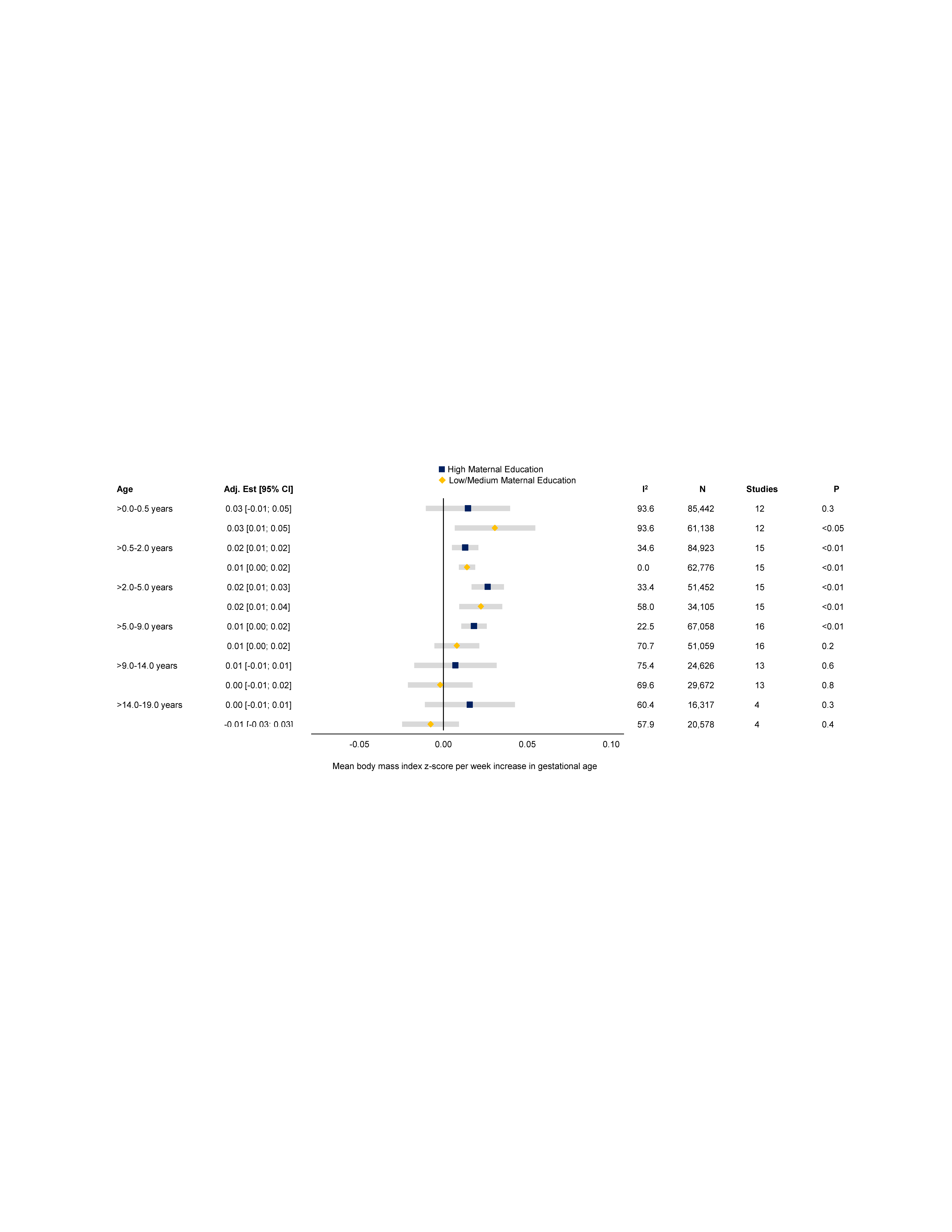

Supplement: S5 Fig — Overall adjusted estimates of BMI z-score with 95% CIs from study-specific linear regression models, where cohorts were assigned weights under the random-effects model to attain the estimate. The blue (high educational level) and yellow (low/medium educational level) dots in the forest plot represent the adjusted estimates, while the whiskers span the 95% CI, whereas I2-statistics (I2), sample size (N), studies, and p-value (two-sided, <0.05) relate to adjusted estimates. Estimates reflect mean differences in BMI z-score per week increase in GA at birth in early infancy (>0.0–0.5 years), late infancy (>0.5–2.0 years), early childhood (>2.0–5.0 years), mid-childhood (>5.0–9.0 years), late childhood (>9.0–14.0 years), and adolescence (>14.0–19.0 years). Models are adjusted for sex of child, and the following maternal characteristics: age at child’s birth, education, height, prepregnancy BMI, smoking during pregnancy, parity, ethnic background, gestational diabetes and hypertension, and preeclampsia. Cohort-specific estimates were adjusted for the maximum available set of the confounding variables (see Table 1). BMI, body mass index; CI, confidence interval; GA, gestational age. (TIF) [file pmed.1004036.s005.tif]

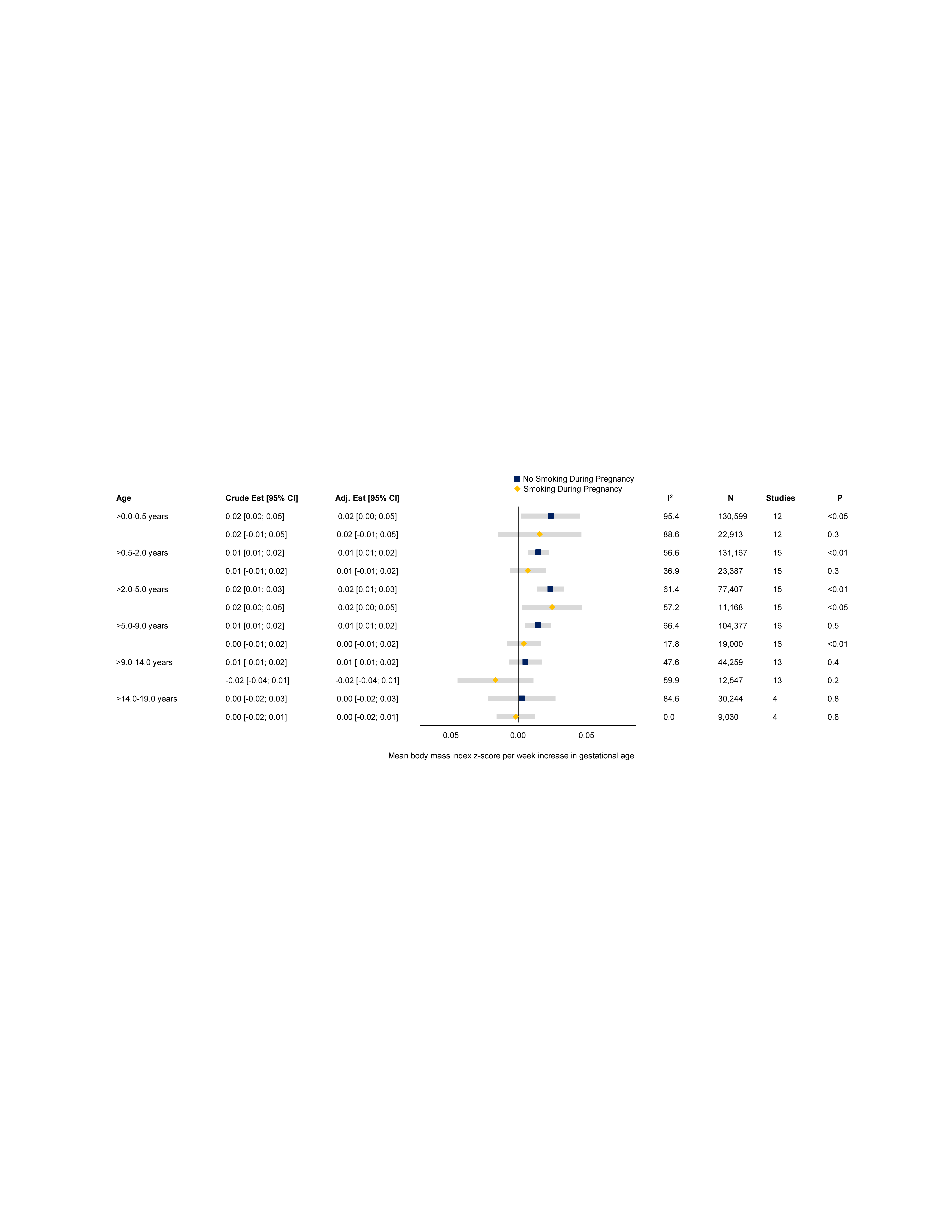

Supplement: S6 Fig — Overall adjusted estimates of BMI z-score with 95% CIs from study-specific linear regression models, where cohorts were assigned weights under the random-effects model to attain the estimate. The blue (smoking in pregnancy) and yellow (no smoking in pregnancy) dots in the forest plot represent the adjusted estimates, while the whiskers span the 95% CI, whereas I2-statistics (I2), sample size (N), studies, and p-value (two-sided, <0.05) relate to adjusted estimates. Estimates reflect mean differences in BMI z-score per week increase in GA at birth in early infancy (>0.0–0.5 years), late infancy (>0.5–2.0 years), early childhood (>2.0–5.0 years), mid-childhood (>5.0–9.0 years), late childhood (>9.0–14.0 years), and adolescence (>14.0–19.0 years). Models are adjusted for sex of child, and the following maternal characteristics: age at child’s birth, education, height, prepregnancy BMI, smoking during pregnancy, parity, ethnic background, gestational diabetes and hypertension, and preeclampsia. Cohort-specific estimates were adjusted for the maximum available set of the confounding variables (see Table 1). BMI, body mass index; CI, confidence interval; GA, gestational age. (TIF) [file pmed.1004036.s006.tif]

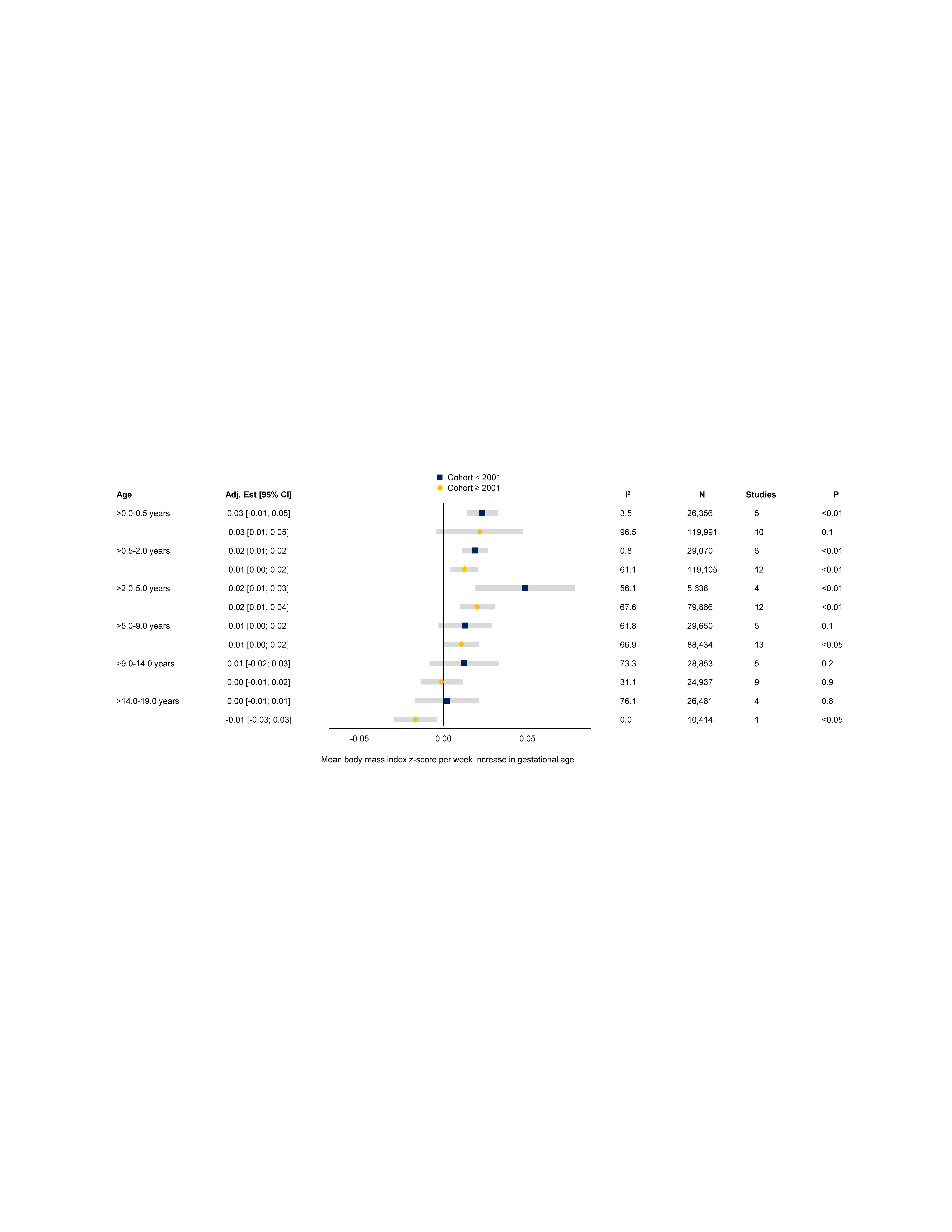

Supplement: S7 Fig — Overall adjusted estimates of BMI z-score with 95% CIs from study-specific linear regression models, where cohorts were assigned weights under the random-effects model to attain the estimate. The blue (<2001) and yellow (≥2001) dots in the forest plot represent the adjusted estimates, while the whiskers span the 95% CI, whereas I2-statistics (I2), sample size (N), studies, and p-value (two-sided, <0.05) relate to adjusted estimates. Estimates reflect mean differences in BMI z-score per week increase in GA at birth in early infancy (>0.0–0.5 years), late infancy (>0.5–2.0 years), early childhood (>2.0–5.0 years), mid-childhood (>5.0–9.0 years), late childhood (>9.0–14.0 years), and adolescence (>14.0–19.0 years). Models are adjusted for sex of child, and the following maternal characteristics: age at child’s birth, education, height, prepregnancy BMI, smoking during pregnancy, parity, ethnic background, gestational diabetes and hypertension, and preeclampsia. Cohort-specific estimates were adjusted for the maximum available set of the confounding variables (see Table 1). BMI, body mass index; CI, confidence interval; GA, gestational age. (TIF) [file pmed.1004036.s007.tif]

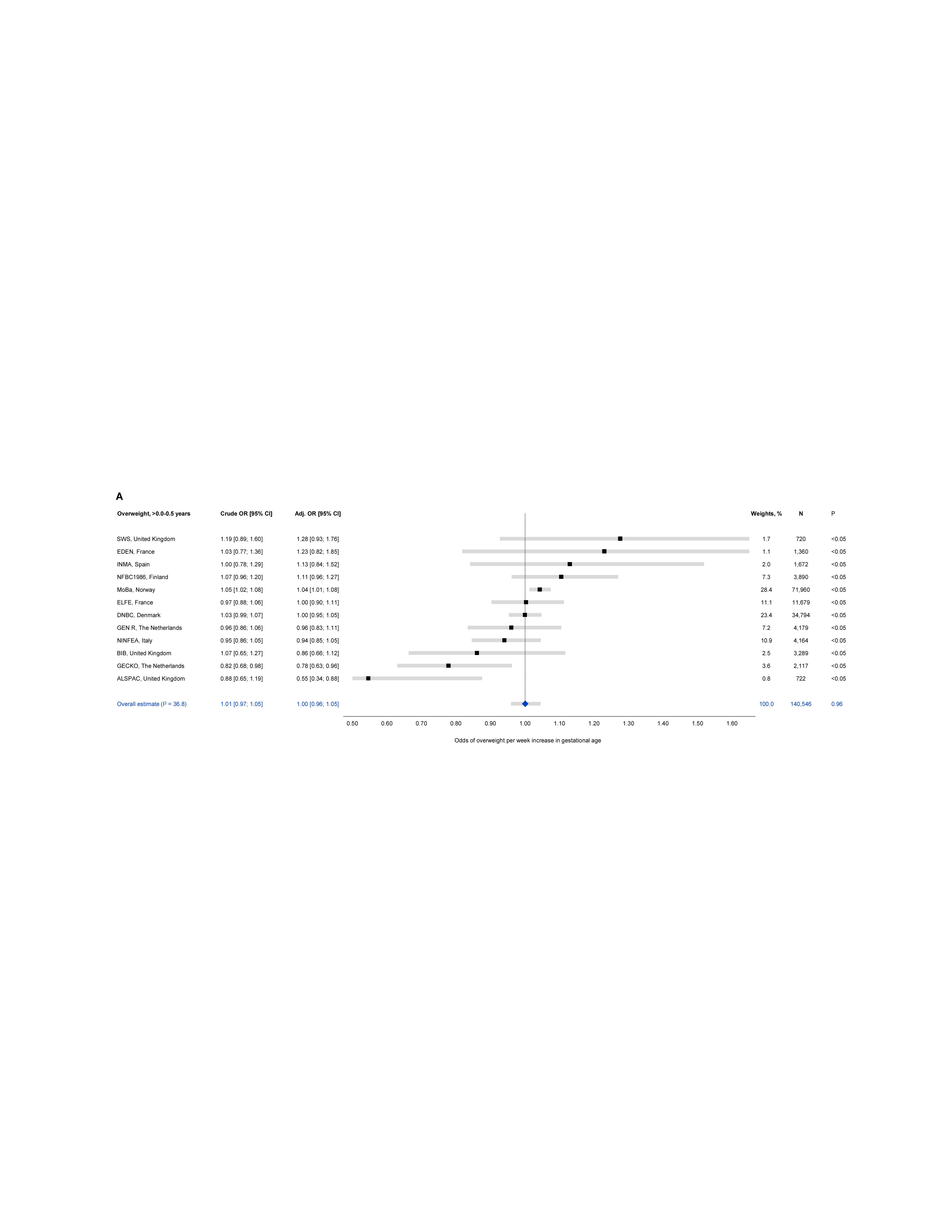

Supplement: S8 Fig — Unadjusted and adjusted ORs with 95% CIs from study-specific logistic regression models, where cohorts were assigned weights under the random-effects model to attain the overall estimates. The dot in the forest plot represents the adjusted OR, while the whiskers span the 95% CI, whereas I2-statistics (I2), sample size (N), studies, and p-value (two-sided, <0.05) relate to adjusted OR. Estimates reflect OR of overweight (vs. normal weight) per week increase in GA at birth in (A) early infancy (>0.0–0.5 years), (B) late infancy (>0.5–2.0 years), (C) early childhood (>2.0–5.0 years), (D) mid-childhood (>5.0–9.0 years), (E) late childhood (>9.0–14.0 years), and (F) adolescence (>14.0–19.0 years). Models are adjusted for sex of child, and the following maternal characteristics: age at child’s birth, education, height, prepregnancy BMI, smoking during pregnancy, parity, ethnic background, gestational diabetes and hypertension, and preeclampsia. Cohort-specific estimates were adjusted for the maximum available set of the confounding variables (see Table 1). BMI, body mass index; CI, confidence interval; GA, gestational age; OR, odds ratio. (TIF) [file pmed.1004036.s008.tif]

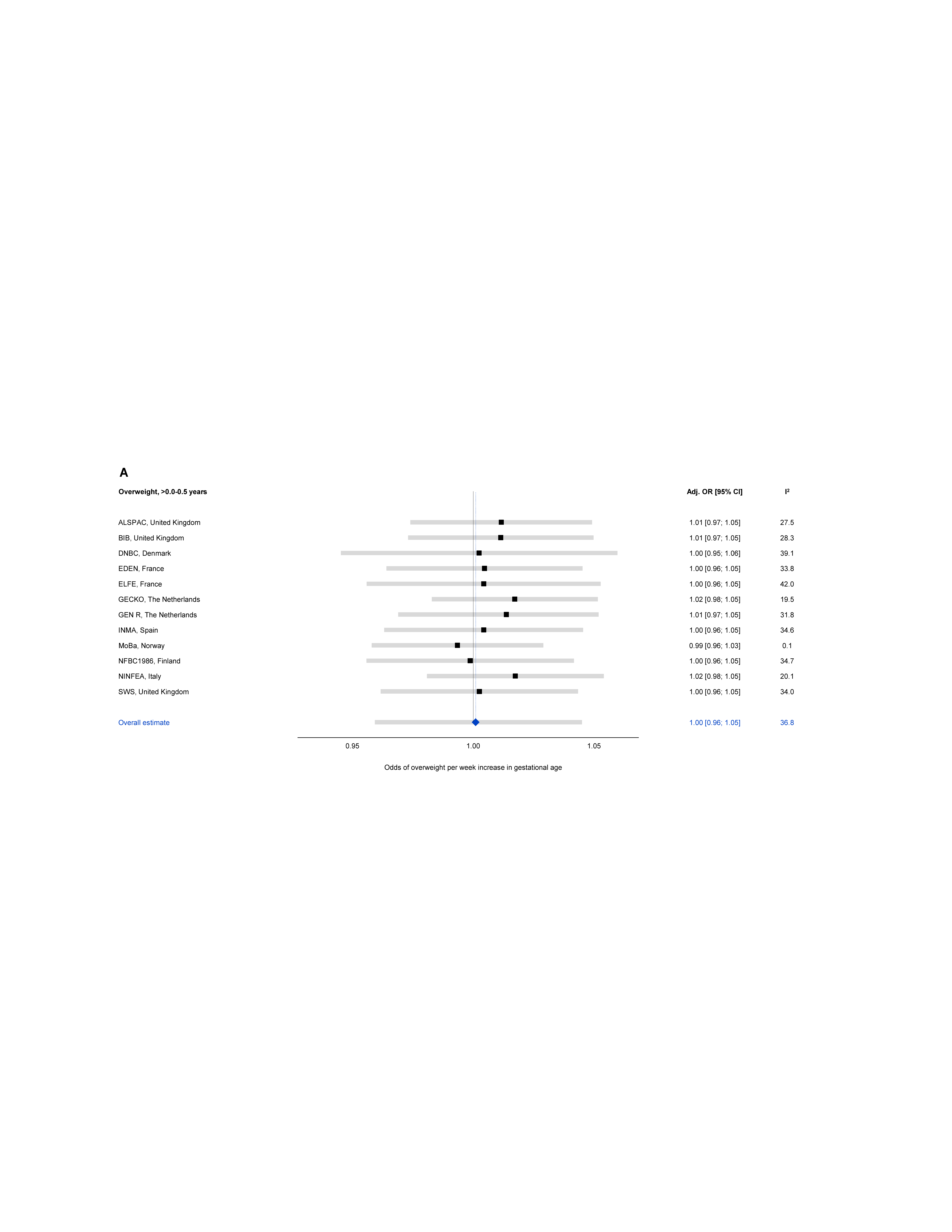

Supplement: S9 Fig — Adjusted ORs with 95% CIs from study-specific logistic regression models, where cohorts were assigned weights under the random-effects model to attain the overall estimates. The dot in the forest plot represents the adjusted OR, while the whiskers span the 95% CI, whereas I2-statistics (I2), sample size (N), studies, and p-value (two-sided, <0.05) relate to adjusted OR. Estimates reflect OR of overweight (vs. normal weight) per week increase in GA at birth in (A) early infancy (>0.0–0.5 years), (B) late infancy (>0.5–2.0 years), (C) early childhood (>2.0–5.0 years), (D) mid-childhood (>5.0–9.0 years), (E) late childhood (>9.0–14.0 years), and (F) adolescence (>14.0–19.0 years). Models are adjusted for sex of child, and the following maternal characteristics: age at child’s birth, education, height, prepregnancy BMI, smoking during pregnancy, parity, ethnic background, gestational diabetes and hypertension, and preeclampsia. Cohort-specific estimates were adjusted for the maximum available set of the confounding variables (see Table 1). BMI, body mass index; CI, confidence interval; GA, gestational age; OR, odds ratio. (TIF) [file pmed.1004036.s009.tif]

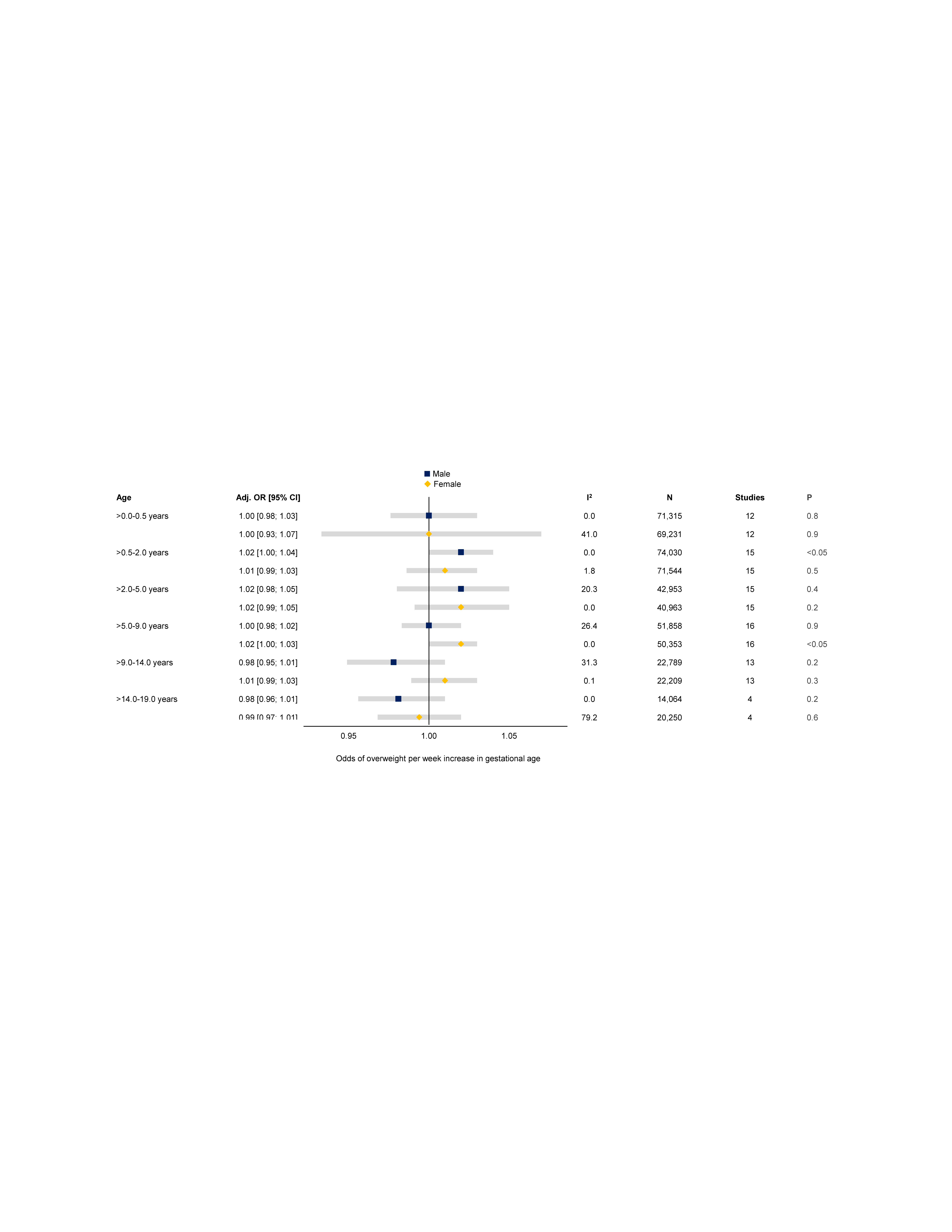

Supplement: S10 Fig — Overall adjusted ORs of overweight with 95% CIs from study-specific linear regression models, where cohorts were assigned weights under the random-effects model to attain the OR. The blue (male) and yellow (female) dots in the forest plot represent the adjusted ORs, while the whiskers span the 95% CI, whereas I2-statistics (I2), sample size (N), studies, and p-value (two-sided, <0.05) relate to adjusted OR. Estimates reflect OR of overweight (vs. normal weight) per week increase in GA at birth in early infancy (>0.0–0.5 years), late infancy (>0.5–2.0 years), early childhood (>2.0–5.0 years), mid-childhood (>5.0–9.0 years), late childhood (>9.0–14.0 years), and adolescence (>14.0–19.0 years). Models are adjusted for sex of child, and the following maternal characteristics: age at child’s birth, education, height, prepregnancy BMI, smoking during pregnancy, parity, ethnic background, gestational diabetes and hypertension, and preeclampsia. Cohort-specific estimates were adjusted for the maximum available set of the confounding variables (see Table 1). BMI, body mass index; CI, confidence interval; GA, gestational age; OR, odds ratio. (TIF) [file pmed.1004036.s010.tif]

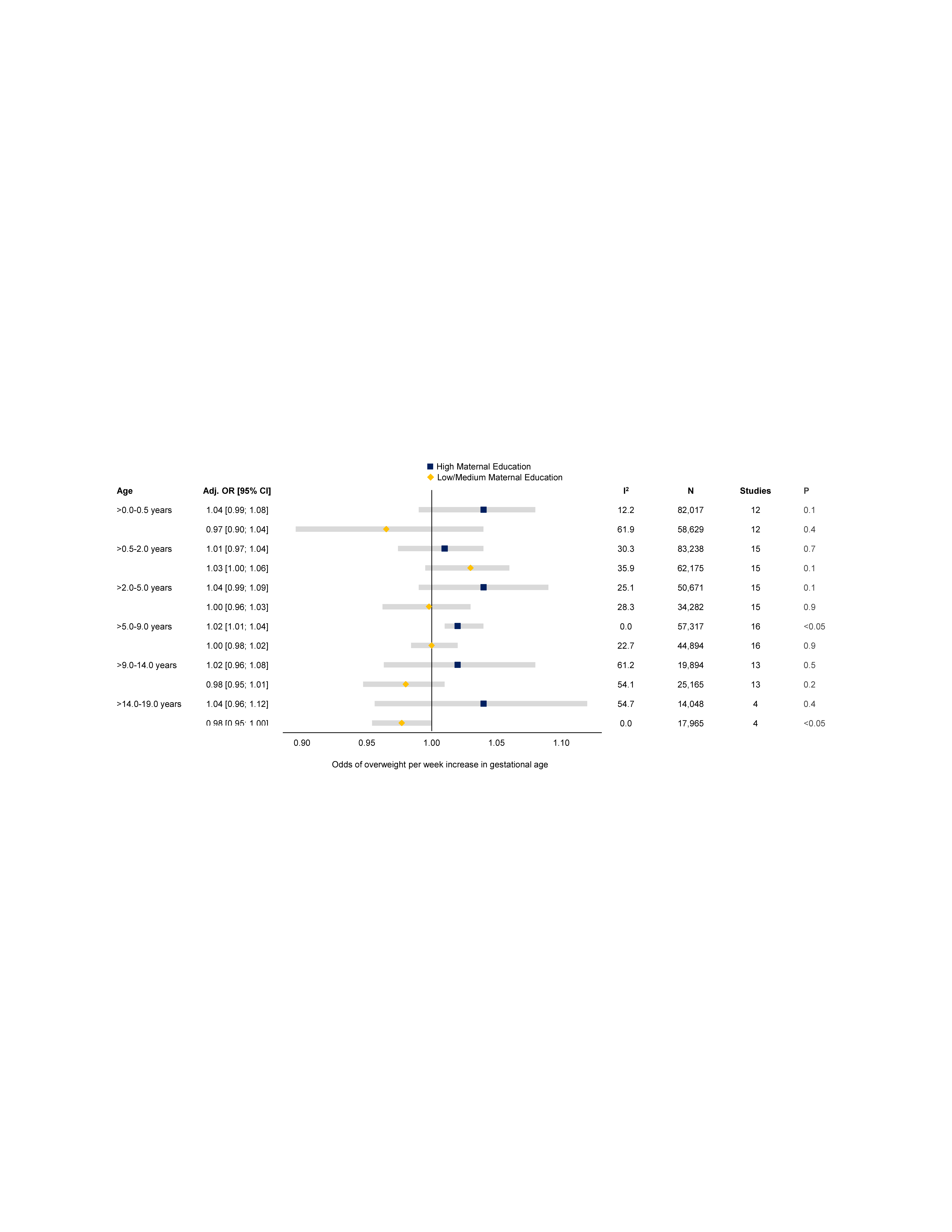

Supplement: S11 Fig — Overall adjusted ORs of overweight with 95% CIs from study-specific linear regression models, where cohorts were assigned weights under the random-effects model to attain the OR. The blue (high educational level) and yellow (low/medium educational level) dots in the forest plot represents the adjusted OR, while the whiskers span the 95% CI, whereas I2-statistics (I2), sample size (N), studies, and p-value (two-sided, <0.05) relate to adjusted OR. Estimates reflect OR of overweight (vs. normal weight) per week increase in GA at birth in early infancy (>0.0–0.5 years), late infancy (>0.5–2.0 years), early childhood (>2.0–5.0 years), mid-childhood (>5.0–9.0 years), late childhood (>9.0–14.0 years), and adolescence (>14.0–19.0 years). Models are adjusted for sex of child, and the following maternal characteristics: age at child’s birth, education, height, prepregnancy BMI, smoking during pregnancy, parity, ethnic background, gestational diabetes and hypertension, and preeclampsia. Cohort-specific estimates were adjusted for the maximum available set of the confounding variables (see Table 1). BMI, body mass index; CI, confidence interval; GA, gestational age; OR, odds ratio. (TIF) [file pmed.1004036.s011.tif]

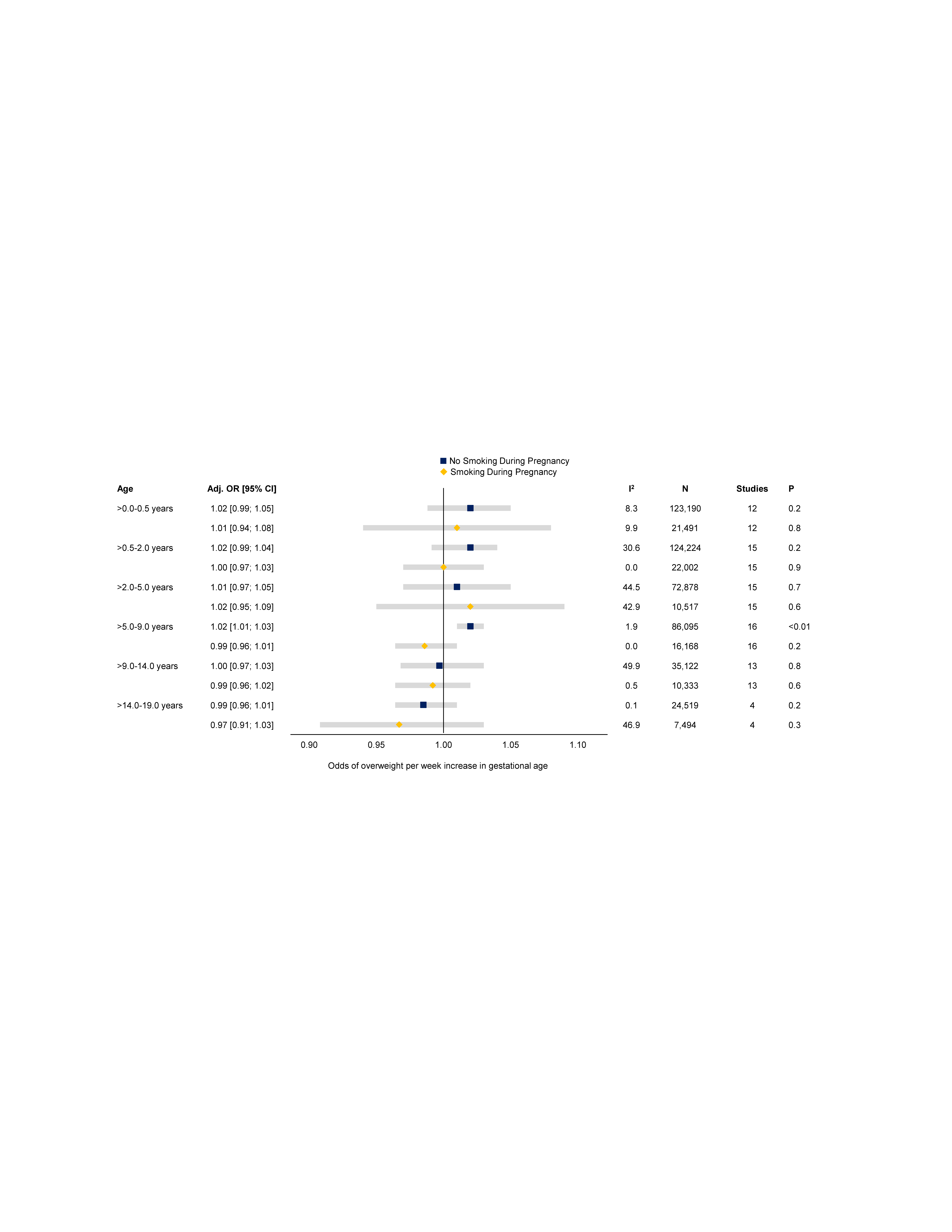

Supplement: S12 Fig — Overall adjusted ORs of overweight with 95% CIs from study-specific linear regression models, where cohorts were assigned weights under the random-effects model to attain the OR. The blue (smoking in pregnancy) and yellow (no smoking in pregnancy) dots in the forest plot represents the adjusted OR, while the whiskers span the 95% CI, whereas I2-statistics (I2), sample size (N), studies, and p-value (two-sided, <0.05) relate to adjusted OR. Estimates reflect OR of overweight (vs. normal weight) per week increase in GA at birth in early infancy (>0.0–0.5 years), late infancy (>0.5–2.0 years), early childhood (>2.0–5.0 years), mid-childhood (>5.0–9.0 years), late childhood (>9.0–14.0 years), and adolescence (>14.0–19.0 years). Models are adjusted for sex of child, and the following maternal characteristics: age at child’s birth, education, height, prepregnancy BMI, smoking during pregnancy, parity, ethnic background, gestational diabetes and hypertension, and preeclampsia. Cohort-specific estimates were adjusted for the maximum available set of the confounding variables (see Table 1). BMI, body mass index; CI, confidence interval; GA, gestational age; OR, odds ratio. (TIF) [file pmed.1004036.s012.tif]

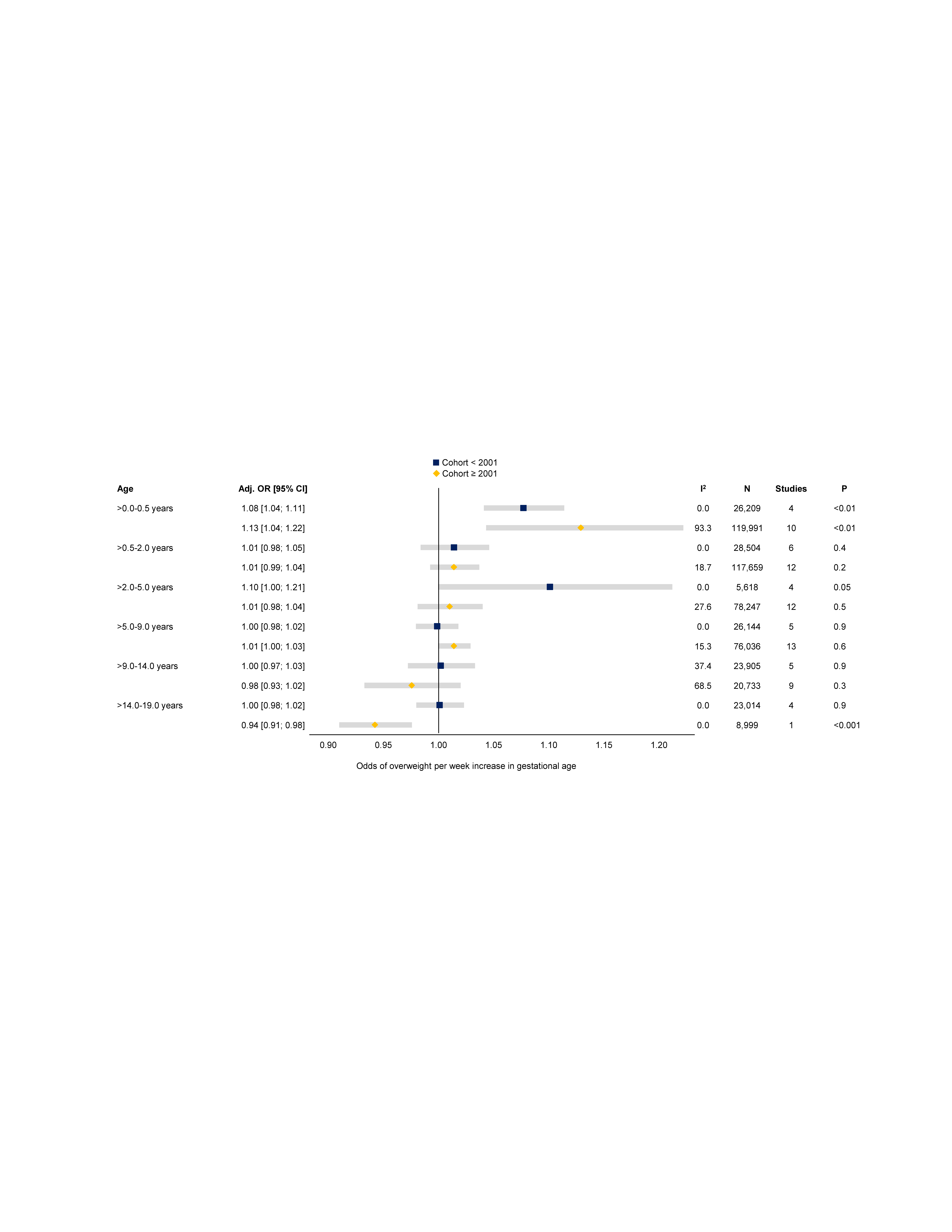

Supplement: S13 Fig — Overall adjusted ORs of overweight with 95% CIs from study-specific linear regression models, where cohorts were assigned weights under the random-effects model to attain the OR. The blue (<2001) and yellow (≥2001) dots in the forest plot represents the adjusted OR, while the whiskers span the 95% CI, whereas I2-statistics (I2), sample size (N), studies, and p-value (two-sided, <0.05) relate to adjusted OR. Estimates reflect OR of overweight (vs. normal weight) per week increase in GA at birth in early infancy (>0.0–0.5 years), late infancy (>0.5–2.0 years), early childhood (>2.0–5.0 years), mid-childhood (>5.0–9.0 years), late childhood (>9.0–14.0 years), and adolescence (>14.0–19.0 years). Models are adjusted for sex of child, and the following maternal characteristics: age at child’s birth, education, height, prepregnancy BMI, smoking during pregnancy, parity, ethnic background, gestational diabetes and hypertension, and preeclampsia. Cohort-specific estimates were adjusted for the maximum available set of the confounding variables (see Table 1). BMI, body mass index; CI, confidence interval; GA, gestational age; OR, odds ratio. (TIF) [file pmed.1004036.s013.tif]
