# Supplementary material for: Gestational age at birth and body size from infancy through adolescence: An individual participant data meta-analysis on 253,810 singletons in 16 birth cohort studies
Source: PLoS Med. 2023 Jan 26;20(1):e1004036. doi: 10.1371/journal.pmed.1004036 (PMC9879424; doi:10.1371/journal.pmed.1004036)
Supplement: S1 Table — (DOCX) [file pmed.1004036.s014.docx]

**S1 Table.** Cohort-specific study characteristics and information on exposure and outcome measurements

| Cohort | Cover Area | Timing of Recruitment  (note) | Source of Delivery Information | Source of Anthropometrics |
| --- | --- | --- | --- | --- |
| ALSPAC^1,2,3^ United Kingdom | City of Bristol and  surrounding areas of  Southwest England | In pregnancy  (median: 14 weeks) | A combination of Last Menstrual Period and Ultra-Sound. Mothers reported the date of their last menstrual period on entry to the study, from which gestational age was calculated. Where there was conflicting information regarding last menstrual period from clinical or pediatric estimates, the clinical records were reviewed, and dating was based on the earliest ultrasound scan. | Weight and height measures were obtained from numerous sources from birth to 18 years, including from midwives, health visitors, linkage to child health records, and ALSPAC research clinics. Birth weight/length data were available from obstetric records. Child health records were used for measures up to 4 years. ALSPAC research clinics were used for measures from 4-18 years |
| AOF Canada | City of Calgary and Alberta | In pregnancy  (< 24 weeks) | A combination of Last Menstrual Period  and Ultra-Sound | Data are collected and reported by mothers through self-report questionnaires during pregnancy, post-partum, and post-birth (1-8 years) |
| BiB United Kingdom | City of Bradford | In pregnancy  (~26 weeks) | A combination of Last Menstrual Period  and Ultra-Sound | Maternity records; BiB cohort and sub-cohort studies; Healthy Child Programme; GP, National Child Measurement Programme |
| CHILD Canada | 4 urban centers in Canada (Vancouver, Edmonton, Winnipeg, Toronto) and 1 rural site (Morden/Winkler Manitoba) | In pregnancy  (~18 weeks) | A combination of Last Menstrual Period  and Ultra-Sound | Measured at clinical examinations by trained research personnel (3 months, 1 year, 3 year, 5 year and 8 year) using standardized assessment tools and protocol. |
| DNBC^4,5,6^ Denmark | Denmark | In pregnancy  (16 weeks) | A combination of Last Menstrual Period  and Ultra-Sound | Reported by parents from routine visits at GP (5 & 12 months, and 7 years) and self-reported (11 years) |
| EDEN France | Poitiers and Nancy | In pregnancy  (<24 weeks) | A combination of Last Menstrual Period  and Ultra-Sound | Measured at clinical examinations (birth to 5 years) and collected from GP repots on child's health booklets |
| ELFE France | France (Metropolitan) | At birth | A combination of Last Menstrual Period  and Ultra-Sound | Collected from GP reports on child's health booklets |
| G21 Portugal | Metropolitan Area of Porto | At birth | A combination of Last Menstrual Period  and Ultra-Sound | Measured by Trained Researchers (all ages) |
| GECKO The Netherlands | Province of Drenthe | In pregnancy  (3rd trimester) | A combination of Last Menstrual Period  and Ultra-Sound | Measured by Youth Health Services (all ages) |
| GEN R The Netherlands | City of Rotterdam | In pregnancy  (<18 weeks, 69%) until birth | Ultra-sound for participants prenatally included; Medical records or self-recorded for participants postnatally included | Measured at routine visits in community child health centres (2-48 months), and at GEN R Research Center (6 & 10 years) |
| INMA ^7^ Spain | 3 areas of Spain (Gipuzkoa, Sabadell, Valencia) | In pregnancy  (10-13 weeks) | A combination of Last Menstrual Period  and Ultra-Sound | Measured in a clinical setting |
| MoBa ^8^ Norway | Norway | In pregnancy  (17-18 weeks)^4^ | Ultra-Sound | Parent-report based on health cards (6 weeks, 3-18 months), and parent-reported (2-8 years) |
| NFBC86 ^9^ Finland | Provinces of Oulu and Lapland | In pregnancy | A combination of Last Menstrual Period (58%),  Ultra-Sound (40%) and hospital records (2%)^5^ | Measured from antenatal cards, school nurse records, and questionnaires |
| NINFEA Italy | Italy | In pregnancy | A combination of Last Menstrual Period  and Ultra-Sound | Reported by parents |
| The Raine Study Australia | Metropolitan Area of Perth | In pregnancy  (16-18 weeks) | A combination of Last Menstrual Period and Ultra-Sound | Measured in a clinical setting (all ages) |
| SWS ^10^ United Kingdom | City of Southampton  and surrounding area | Before pregnancy  (median: -130 weeks) | A combination of Last Menstrual Period and Ultra-Sound | Measurements of children by research nurses ( at birth,v6, 12, 24 months, 3-4 years, 6 years, 8 years) Measurements of mothers by research nurses (pre-pregnancy, around 11 and 34 weeks of gestation) |

**Reference**

1. Iles-Caven, Y., Northstone, K., & Golding, J. Gestation at completion of prenatal questionnaires in ALSPAC.  Wellcome open research 2020; 5, 100.
2. Nicola J. Wiles, Tim J. Peters, Jon Heron, David Gunnell, Alan Emond, Glyn Lewis, for the ALSPAC Study Team. Fetal Growth and Childhood Behavioral Problems: Results from the ALSPAC Cohort. Am J Epidemiol. 2006;163(9):829–837
3. Howe LD, Tilling K, Lawlor DA. Accuracy of height and weight data from child health records. Arch Dis Child. 2009 Dec;94(12):950-954
4. Olsen J, Melbye M, Olsen SF, Sorensen TI, Aaby P, Andersen AM, et al. The Danish National Birth Cohort - its background, structure and aim. Scand J Public Health. 2001; 29(4):300-307.
5. Morgen CS, Andersen PK, Mortensen LH, Howe LD, Rasmussen M, Due P, et al. Socioeconomic disparities in birth weight and body mass index during infancy through age 7 years: a study within the Danish National Birth Cohort. BMJ Open. 2017:20: 7(1):e011781.
6. Morgen CS, Ängquist L, Baker JL, Andersen AMN, Sørensen TIA, Michaelsen KF. Breastfeeding and complementary feeding in relation to body mass index and overweight at ages 7 and 11 y: a path analysis within the Danish National Birth Cohort. The American Journal of Clinical Nutrition. 2018: 107(3):313-322
7. Guxens M, Ballester F, Espada M, Fernández MF, Grimalt JO, Ibarluzea J, et al. INMA Project. Cohort Profile: the INMA--INfancia y Medio Ambiente--(Environment and Childhood) Project. Int J Epidemiol. 2012 41(4):930-940
8. Schreuder P, Alsaker E. The Norwegian Mother and Child Cohort Study (MoBa) – MoBa recruitment and logistics. Norsk Epidemiologi 2014; 24 (1-2): 23-27
9. Sipola-Leppänen M, Vääräsmäki M, Tikanmäki M, Hovi P, Miettola S, Ruokonen A, et al. Cardiovascular risk factors in adolescents born preterm. Pediatrics 2014; 134(4):e1072
10. Inskip HM, Godfrey KM, Robinson SM, Law CM, Barker DJ, Cooper C, et al. Cohort profile: The Southampton Women's Survey. Int J Epidemiol. 2006;35(1):42-48
