# Supplementary material for: Gestational age at birth and body size from infancy through adolescence: An individual participant data meta-analysis on 253,810 singletons in 16 birth cohort studies
Source: PLoS Med. 2023 Jan 26;20(1):e1004036. doi: 10.1371/journal.pmed.1004036 (PMC9879424; doi:10.1371/journal.pmed.1004036)
Supplement: S2 Table — (DOCX) [file pmed.1004036.s015.docx]

**S2 Table**. Missing values in cohort-specific baseline characteristics

|  | *N* | Maternal Age at Child's Birth | Maternal  Education | Maternal  Ethnicity | Maternal  Height | Pre-pregnancy BMI | Smoking in Pregnancy | Gestational Diabetes | Gestational Hypertension | Maternal Preeclampsia | Parity |
| --- | --- | --- | --- | --- | --- | --- | --- | --- | --- | --- | --- |
| ALSPAC United Kingdom | *10,452* | 917 (8.8) | 557 (5.3) | 652 (6.2) | 728 (7.0) | 1,606 (15.4) | 1,204 (11.5) | 600 (5.7) | 246 (2.4) | 46 (0.4) | 449 (4.3) |
| AOF Canada | *2,263* | 0 (0.0) | 5 (0.2) | 10 (0.4) | 11 (0.5) | 30 (1.3) | 0 (0.0) | 0 (0.0) | 0 (0.0) | 0 (0.0) | 19 (0.8) |
| BiB United Kingdom | *13,097* | 0 (0.0) | 3,120 (23.8) | 2,257 (17.2) | 2,473 (18.9) | 8,451 (64.5) | 2,266 (17.3) | 86 (6.6) | 2,394 (18.3) | 2,394 (18.3) | 485 (3.7) |
| CHILD Canada | *2,984* | 0 (0.0) | 101 (3.4) | 27 (0.9) | 93 (3.1) | 949 (31.8) | 70 (2.3) | 0 (0.0) | 20,505 (16.3) | 70 (2.3) | 2 (0.1) |
| DNBC Denmark | *81,117* | 0 (0.0) | 9,002 (11.1) |  | 4,034 (5.0) | 5,186 (6.4) | 1,182 (1.5) | 0 (0.0) | 2 (25.3) | 133 (1.6) | 0 (0.0) |
| EDEN France | *1,765* | 0 (0.0) | 11 (0.6) | 244 (13.8) | 25 (1.4) | 36 (2.0) | 5 (0.3) | 2 (0.1) | 276 (0.1) | 2 (1.1) | 3 (0.2) |
| ELFE France | *15,506* | 49 (0.3) | 3 (0.0) | 1,034 (6.7) | 47 (0.3) | 197 (1.3) | 136 (0.9) | 612 (3.9) | 32 (1.8) | 276 (1.8) | 193 (1.2) |
| G21 Portugal | *6,439* | 4 (0.1) | 36 (0.6) | 332 (5.2) | 13 (0.2) | 127 (2.0) | 2,820 (43.8) | 32 (0.5) | 72 (0.5) | 0 (0.0) | 114 (1.8) |
| GECKO The Netherlands | *2,768* | 24 (0.9) | 193 (7.0) | 189 (6.8) | 147 (5.3) | 234 (8.5) | 29 (1.0) | 383 (13.8) | 1,045 (2.6) | 228 (8.2) | 41 (1.5) |
| GEN R The Netherlands | *8,641* | 0 (0.0) | 893 (10.3) | 467 (5.4) | 317 (3.7) | 2,025 (23.4) | 1,212 (14.0) | 343 (3.9) | 1,936 (12.1) | 1,045 (12.1) | 242 (2.8) |
| INMA Spain | *1,936* | 0 (0.0) | 39 (2.0) | 35 (1.8) | 17 (0.9) | 17 (0.9) | 24 (1.2) | 138 (7.1) |  |  | 88 (4.5) |
| MoBa Norway | *86,553* | 136 (0.2) | 5,109 (5.9) |  | 1,861 (2.2) | 3,061 (3.5) | 48 (0.0) | 973 (1.1) | 3,528 (4.1) | 3,528 (4.1) | 973 (1.1) |
| NFBC1986 Finland | *8,325* | 0 (0.0) | 1,027 (12.3) |  | 65 (0.8) | 183 (2.2) | 45 (0.5) |  | 1,590 (19.1) | 1,691 (20.3) | 26 (0.3) |
| NINFEA Italy | *6,514* | 0 (0.0) | 47 (0.7) |  | 135 (2.1) | 144 (2.2) | 76 (1.2) | 575 (8.8) | 549 (8.4) | 536 (8.2) | 314 (4.8) |
| The Raine study Australia | *2,443* | 4 (0.2) | 121 (0.5) | 0 (0.0) | 0 (0.0) | 68 (2.8) | 0 (0.0) | 0 (0.0) | 66 (2.7) | 66 (2.7) | 0 (0.0) |
| SWS United Kingdom | *3,007* | 0 (0.0) | 9 (0.3) | 1 (0.3) | 16 (0.5) | 27 (0.9) | 137 (4.6) | 0 (0.0) | 0 (0.0) | 0 (0.0) | 3 (0.1) |

**Note**: Empty cells represent no available data

**Abbreviations**: N = Sample Size, BMI = Body Mass Index
