# Supplementary material for: Gestational age at birth and body size from infancy through adolescence: An individual participant data meta-analysis on 253,810 singletons in 16 birth cohort studies
Source: PLoS Med. 2023 Jan 26;20(1):e1004036. doi: 10.1371/journal.pmed.1004036 (PMC9879424; doi:10.1371/journal.pmed.1004036)
Supplement: S3 Table — (DOCX) [file pmed.1004036.s016.docx]

**S3 Table**. Results of individual variable meta-regression models showing values of β, se(β), and the significance of β for each study characteristic

| **Age-band** | **Study Characteristics** | **β** | **se(β)** | **p-value (<0.05, two-sided)** |
| --- | --- | --- | --- | --- |
| Early infancy (>0.0-0.5 years) | *Age at measurement*, months | -0.0296 | 0.0076 | <0.01 |
|  | *Sex*, female (%) | 0.0016 | 0.0096 | 0.87 |
|  | *Maternal education*, low (%) | 0.0011 | 0.0006 | 0.10 |
|  | *Maternal smoking in pregnancy*, yes (%) | -0.0005 | 0.0011 | 0.68 |
| Mid-childhood (>5.0-9.0 years) | *Age at measurement*, months | 0.0006 | 0.0005 | 0.27 |
|  | *Sex*, female (%) | 0.0042 | 0.0047 | 0.38 |
|  | *Maternal education*, low (%) | 0.0002 | 0.0003 | 0.59 |
|  | *Maternal smoking in pregnancy*, yes (%) | 0.0000 | 0.0005 | 0.94 |
| Late childhood (>9.0-14.0 years) | *Age at measurement*, months | 0.0001 | 0.0005 | 0.83 |
|  | *Sex*, female (%) | 0.0019 | 0.0071 | 0.77 |
|  | *Maternal education*, low (%) | 0.0009 | 0.0005 | 0.05 |
|  | *Maternal smoking in pregnancy*, yes (%) | 0.0002 | 0.0007 | 0.74 |
| Adolescence (>14.0-19.0 years) | *Age at measurement*, months | -0.0007 | 0.0012 | 0.58 |
|  | *Sex*, female (%) | -0.0020 | 0.0034 | 0.55 |
|  | *Maternal education*, low (%) | 0.0012 | 0.0005 | <0.01 |
|  | *Maternal smoking in pregnancy*, yes (%) | 0.0074 | 0.0030 | <0.05 |

**Abbreviations**: β = beta-coefficient, se(β) = standard error of beta-coefficient
