# Supplementary material for: Gestational age at birth and body size from infancy through adolescence: An individual participant data meta-analysis on 253,810 singletons in 16 birth cohort studies
Source: PLoS Med. 2023 Jan 26;20(1):e1004036. doi: 10.1371/journal.pmed.1004036 (PMC9879424; doi:10.1371/journal.pmed.1004036)
Supplement: S1 Text — (DOCX) [file pmed.1004036.s017.docx]

**S1 Text**. Information about variable classification and coding

**Gestational Age at Birth (GA)**

Each cohort decided the most accurate available measure of GA, but a priority was given to last menstrual period (LMP), unless it varied from an ultrasound-based (US) estimate by more than 1 week, in which ultrasound was used. If LMP was not available, US was used. If LMP or US was not available, information from a maternal report was used.

**Maternal Education**

Maternal education was classified according to International Classification of Education 97/2011 (ISCED 97/2011) as either *high, medium* or *low.* High includes short cycle tertiary, Bachelor, Masters, Doctoral or equivalent (ISCED-2011: 5-8, ISCED-97: 5-6); Medium includes Upper Secondary, Post-secondary non-tertiary (ISCED-2011: 3-4, ISCED-97: 3-4); Low includes No education, early childhood, pre-primary, primary, lower secondary or second stage of basic education (ISCED-2011: 0-2, ISCED-97: 0-2).

**Maternal Ethnic Background**

Maternal ethnic background was based on (a) *colour of mother* (white/Caucasian, non-white/non-Caucasian) or (b) *country of origin of parents* (western, non-western or mixed), where western countries include European Union, Andorra, Australia, Canada, Iceland, Liechtenstein, Monaco, New Zealand, Norway, San Marino, Switzerland, USA and Vatican City. Non-western countries include all other countries, while mixed refers to one parent from a western country and one parent from a non-western country.

The distinction between and definition of western and non-western is chosen in accordance with Statistics Denmark (<https://www.dst.dk/Site/Dst/SingleFiles/GetArchiveFile.aspx?fi=91448101625&fo=0&ext=kvaldel>, guidelines, which is a continuation of a former UN-definition
