# Supplementary material for: Gestational age at birth and body size from infancy through adolescence: An individual participant data meta-analysis on 253,810 singletons in 16 birth cohort studies
Source: PLoS Med. 2023 Jan 26;20(1):e1004036. doi: 10.1371/journal.pmed.1004036 (PMC9879424; doi:10.1371/journal.pmed.1004036)
Supplement: S1 Appendix — (DOCX) [file pmed.1004036.s018.docx]

**S1 Appendix**. Cohort-specific sources of funding/support

**ALSPAC**

Core funding for the Avon Longitudinal Study of Parents and Children (ALSPAC) is provided by the UK Medical Research Council and Wellcome (217065/Z/19/Z) and the University of Bristol. A comprehensive list of grants funding is available on the ALSPAC website (http://www.bristol.ac.uk/alspac/external/documents/grant-acknowledgements.pdf). DAL and AK work in a unit that is supported by the University of Bristol and UK Medical Research Council (MC_UU_00011/6) and DAL holds a European Research Council Advanced Grant (ERC grant agreement no 669545) and is a NIHR Senior Investigator (NF-0616-10102). The funders had no role in the design of the study, the collection, analysis, or interpretation of the data; the writing of the manuscript, or the decision to submit the manuscript for publication. The views expressed in this paper are those of the authors and not necessarily those of any funder.

**AOF**

All Our Families is funded through Alberta Innovates Interdisciplinary Team Grant #200700595, the Alberta Children’s Hospital Foundation, the Canadian Institutes of Health Research, and the Social Sciences and Humanities Research Council. Initial funding to investigate maternal and infant outcomes during the perinatal period was provided by Three Cheers for the Early Years, Alberta Health Services. Expansion of the cohort, to include collection of biological specimens to predict determinants of adverse birth outcomes, was funded by Alberta Innovates Health Solutions [Interdisciplinary Team Grant #200700595] and the Global Alliance to Prevent Prematurity and Stillbirth [GAPPS award #12006]. Subsequent collaborations focusing on the developmental trajectories of AOB children have been supported by numerous grants, including the Social Sciences and Humanities Research Council, Alberta Health Services, the Alberta Children’s Hospital Foundation, the Alberta Centre for Child, Family and Community Research, Upstart the United Way of Calgary, Women and Children’s Health Research Institute, the Canadian Foundation for Fetal Alcohol Research and the Max Bell Foundation.

**BiB**

BiB receives core infrastructure funding from the Wellcome Trust (WT101597MA) and a joint grant from the UK Medical Research Council (MRC) and Economic and Social Science Research Council (ESRC) (MR/N024397/1). This study has received support from the British Heart Foundation (CS/16/4/32482), US National Institutes of Health (R01 DK10324), European Research Council under the European Union's Seventh Framework Programme (FP7/2007-2013) / ERC grant agreement no 669545, and National Institute for Health Research ARC Yorkshire and Humber (NIHR200166. PMW receives funding from the National Institute for Health Research Applied Research Collaboration for Greater Manchester. The views expressed are those of the author(s), and not necessarily those of the NHS, the NIHR or the Department of Health and Social Care.

**CHILD**

The CHILD Cohort Study was launched in 2008 with funding from AllerGen NCE Inc. and the Canadian Institutes of Health Research (CIHR). Additional funding has been provided for the core study by Genome Canada and local provincial funding and philanthropic support. The funders had no role in the design of the study, the collection, analysis, or interpretation of the data; the writing of the manuscript, or the decision to submit the manuscript for publication. The views expressed in this paper are those of the authors and not necessarily those of any funder

**DNBC**

The Danish National Birth Cohort was established with a significant grant from the Danish National Research Foundation. Additional support was obtained from the Danish Regional Committees, the Pharmacy Foundation, the Egmont Foundation, the March of Dimes Birth Defects Foundation, the Health Foundation and other minor grants. The DNBC Biobank has been supported by the Novo Nordisk Foundation and the Lundbeck Foundation. Follow-up of mothers and children have been supported by the Danish Medical Research Council (SSVF 0646, 271-08-0839/06-066023, O602-01042B, 0602-02738B), the Lundbeck Foundation (195/04, R100-A9193), The Innovation Fund Denmark 0603-00294B (09-067124), the Nordea Foundation (02-2013-2014), Aarhus Ideas (AU R9-A959-13-S804), University of Copenhagen Strategic Grant (IFSV 2012), and the Danish Council for Independent Research (DFF – 4183-00594 and DFF - 4183-00152). AP is funded by a Lundbeck Foundation fellowship (R264-2017-3099)

**EDEN**

The EDEN study was supported by Foundation for medical research (FRM), National Agency for Research (ANR), National Institute for Research in Public health (IRESP: TGIR cohorte santé 2008 program), French Ministry of Health (DGS), French Ministry of Research, INSERM Bone and Joint

Diseases National Research (PRO-A) and Human Nutrition National Research Programs, Paris-Sud University, Nestlé, French National Institute for Population Health Surveillance (InVS), French National Institute for Health Education (INPES), the European Union FP7 programmes (FP7/2007-2013, HELIX, ESCAPE, ENRIECO, Medall projects), Diabetes National Research Program (through a collaboration with the French Association of Diabetic Patients (AFD)), French Agency for Environmental Health Safety (now ANSES), Mutuelle Générale de l’Education Nationale a complementary health insurance (MGEN), French national agency for food security, French speaking association for the study of diabetes and metabolism (ALFEDIAM).

**ELFE**

The Elfe cohort received funding from the National Research Agency Investment for the Future program [ANR-11-EQPX-0038]; French National Institute for Research in Public Health (IRESP TGIR 2009-2001 program); Ministry of Higher Education and Research; Ministry of Environment; Ministry of Health; French Agency for Public Health; Ministry of Culture; and National Family Allowance Fund.

**G21**

Generation XXI was supported by the European Regional Development Fund (ERDF) through the Operational Programme Competitiveness and Internationalization and national funding from the Foundation for Science and Technology (FCT), Portuguese Ministry of Science, Technology and Higher Education under the project “HIneC: When do health inequalities start? Understanding the impact of childhood social adversity on health trajectories from birth to early adolescence” (POCI-01-0145-FEDER-029567; Reference PTDC/SAU-PUB/29567/2017). It is also supported by the Unidade de Investigação em Epidemiologia - Instituto de Saúde Pública da Universidade do Porto (EPIUnit) (UIDB/04750/2020), Administração Regional de Saúde Norte (Regional Department of Ministry of Health) and Fundação Calouste Gulbenkian; PhD Grant SFRH/BD/108742/2015 (to SS) co-funded by FCT and the Human Capital Operational Programme (POCH/FSE Program). This project received funding from the European Union's Horizon 2020 research and innovation programme under the project ; EUCAN-Connect grant agreement No 824989. ACS is founded by a FCT Investigator contracts IF/01060/2015.

**GECKO**

The GECKO Drenthe birth cohort was funded by an unrestricted grant of Hutchison Whampoa Ltd, Hong Kong and supported by the University of Groningen , Well Baby Clinic Foundation Icare, Noordlease, Paediatric Association Of The Netherlands, Youth Preventive Health Care Drenthe and European Union’s Horizon 2020 research and innovation programme (LifeCycle, Grant Agreement No. 733206 LifeCycle).

**Generation R**

The general design of the Generation R Study is made possible by financial support from the Erasmus MC, University Medical Center, Rotterdam, Erasmus University Rotterdam, Netherlands Organization for Health Research and Development (ZonMw), Netherlands Organisation for Scientific Research (NWO), Ministry of Health, Welfare and Sport and Ministry of Youth and Families. This project received funding from the European Union's Horizon 2020 research and innovation programme (LIFECYCLE, grant agreement No 733206, 2016; EUCAN-Connect grant agreement No 824989; ATHLETE, grant agreement No 874583). VJ received funding from a Consolidator Grant from the European Research Council (ERC-2014-CoG-648916). The study sponsors had no role in the study design, data analysis, interpretation of data, or writing of this report.

**INMA**

INMA-Valencia was funded by Grants from UE (FP7-ENV-2011 cod 282957 and HEALTH.2010.2.4.5-1), Spain: ISCIII (G03/176; FIS-FEDER: PI09/02647, PI11/01007, PI11/02591, PI11/02038, PI13/1944, PI13/2032, PI14/00891, PI14/01687, and PI16/1288; Miguel Servet-FEDER CP11/00178, CP15/00025, and CPII16/00051), and Generalitat Valenciana: FISABIO (UGP 15-230, UGP-15-244,and UGP-15-249). INMA-Gipuzkoa was funded by grants from the Instituto de Salud Carlos III (FISFIS PI06/0867, FISPS09/0009) 0867,Red INMA G03/176) and the Departamento de Salud del Gobierno Vasco (2005111093 and 2009111069) and the Provincial Government of Guipúzcoa (DFG06/004 and FG08/001). INMA-Sabadell was funded by grants from ISCIII (Red INMA G03/176; CB06/02/0041; FIS-FEDER: PI041436; PI081151; PI12/01890; CP13/00054; PI15/00118; CP16/00128; PI16/00118; PI16/00261; PI18/00547), CIBERESP, Generalitat de Catalunya-CIRIT (1999SGR 00241), Generalitat de Catalunya-AGAUR (2009 SGR 501, 2014 SGR 822), Fundació La Marató de TV3 (090430), Spanish Ministry of Economy and Competitiveness (SAF2012-32991 incl. FEDER funds), Agence Nationale de Securite Sanitaire de l’Alimentation de l’Environnement et du Travail (1262C0010; EST-2016 RF-21), and the European Commission (261357, 308333, 603794 and 634453). ISGlobal acknowledges support from the Spanih Ministry of Science, Innovation and Universities through the “Centro de Excelencia Severo Ochoa 2019-2023” Program (CEX2018-000806-S), and support from the Generalitat de Catalunya through the CERCA Program.

**MoBa**

The Norwegian Mother, Father and Child Cohort Study is supported by the Norwegian Ministry of Health and Care Services and the Ministry of Education and Research. JN received funding, in part, from the European Union's Horizon 2020 research and innovation programme (LIFECYCLE, grant agreement No 733206), The work of JRH was partly supported by the Research Council of Norway through its Centres of Excellence funding scheme, project number 262700.

**NINFEA**

The NINFEA cohort was initially funded by the Compagnia SanPaolo Foundation and the Piedmont Region. It received funding from European projects: CHICOS (FP7 grant number HEALTH-FP7-2009-241604, LifeCycle (H2020 grant number 733206), ATHLETE (H2020 grant number 874583).

**NFBC1986**

NFBC1986 received financial support from EU QLG1-CT-2000-01643 (EUROBLCS, grant number E51560), NorFA (grant numbers 731, 20056 and 30167) and USA / NIH 2000 G DF682 (grant number 50945). Financial support for data generation, research and supporting staff was received from the Academy of Finland (grant numbers: 104781, 120315, 129269, 1114194, 24300796, 285547 (EGEA)); University Hospital Oulu, Biocenter, University of Oulu, Finland (grant number: 75617); NIHM (grant number: MH063706, Smalley and Jarvelin for NFBC1986 data collection), Juselius Foundation; NIH/NIMH (grant number: 5R01MH63706:02); the European Commission: EURO-BLCS, Framework 5 award QLG1-CT-2000-01643 (for NFBC1986 data collection), ENGAGE project and grant agreement HEALTH-F4-2007 (grant number: 201413); EU H2020-HCO-2004 iHEALTH Action (grant number: 643774), EU H2020-PHC-2014 DynaHealth Action (grant number: 633595); ALEC Action (grant number: 633212); ERDF European Regional Development Fund (grant number: 539/2010 A31592); the Medical Research Council (MRC), UK (grant numbers: G0500539, G0600705, G1002319, MR/M013138/1), EU H2020-SC1-2016-2017 LifeCycle Action (grant number: 733206). The programme is currently funded by EU H2020-SC1-2016-2017 LifeCycle Action (grant number: 733206), EU-H2020 EUCAN Connect (grant number: 824989) and H2020-SC1-2019 LongITools (grant number 874739).

**The Raine study**

The Raine study has been funded by program and project grants from the Australian National Health and Medical Research Council (NHMRC), the Commonwealth Scientific and Industrial Research Organisation, Healthway and the Lions Eye Institute in Western Australia. The Raine study Gen2-17 year follow-up was funded by the NHMRC Program Grant (Stanley et al, ID 353514). The Raine study participation in LIFECYCLE was funded by a grant from the National Health and Medical Research Council, Australia (GNT114285). The University of Western Australia (UWA), Curtin University, the Raine Medical Research Foundation, the Telethon Kids Institute, the Women’s and Infant’s Research Foundation (KEMH), Murdoch University, The University of Notre Dame Australia and Edith Cowan University provide funding for the Core Management of the Raine Study. RCH was supported by NHMRC fellowship (Grant Number 1053384). JC is supported by NHMRC EU (Grant Number 114285).

**SWS**

The SWS is supported by grants from the Medical Research Council, National Institute for Health Research Southampton Biomedical Research Centre, British Heart Foundation, University of Southampton and University Hospital Southampton National Health Service Foundation Trust, and the European Union’s Seventh Framework Programme (FP7/2007-2013), project Early Nutrition (grant 289346) and from the European Union's Horizon 2020 research and innovation programme (LIFECYCLE, grant agreement No 733206). Study participants were drawn from a cohort study funded by the Medical Research Council and the Dunhill Medical Trust.
