# Supplementary material for: Gestational age at birth and body size from infancy through adolescence: An individual participant data meta-analysis on 253,810 singletons in 16 birth cohort studies
Source: PLoS Med. 2023 Jan 26;20(1):e1004036. doi: 10.1371/journal.pmed.1004036 (PMC9879424; doi:10.1371/journal.pmed.1004036)
Supplement: S2 Appendix — (DOCX) [file pmed.1004036.s019.docx]

**S2 Appendix**. Cohort-specific acknowledgments

**ALSPAC**

We are extremely grateful to all of the families who took part in ALSPAC, the midwives for their help in recruiting them, and the whole ALSPAC team, which includes interviewers, computer and laboratory technicians, clerical workers, research scientists, volunteers, managers, receptionists and nurses.

**AOF**

The authors acknowledge the tremendous contribution and support of AOF participants and AOF team members. We are extremely grateful to the investigators, coordinators, research assistants, graduate and undergraduate students, volunteers, clerical staff and managers.

**BiB**

The authors acknowledge that Born in Bradford is only possible because of the enthusiasm and commitment of the children and parents in Born in Bradford. We are grateful to all participants, health professionals and researchers who have made Born in Bradford happen.

**CHILD**

We thank the CHILD Cohort Study (CHILD) participant families for their dedication and commitment to advancing health research. Visit CHILD at childcohort.ca.

**DNBC**

The authors would like to thank the participants, the first Principal Investigator of DNBC Prof. Jørn Olsen, the scientific managerial team, and DNBC secretariat for being, establishing, developing and consolidating the Danish National Birth Cohort.

**EDEN**

The authors thank the cohort participants and the EDEN mother-child study group, whose members are: I. Annesi-Maesano, J.Y. Bernard, J. Botton, M.A. Charles, P. Dargent-Molina, B. de Lauzon-Guillain, P. Ducimetière, M. de Agostini, B. Foliguet, A. Forhan, X. Fritel, A. Germa, V. Goua, R. Hankard, B. Heude, M. Kaminski, B. Larroque†, N. Lelong, J. Lepeule, G. Magnin, L. Marchand, C. Nabet, F Pierre, R. Slama, M.J. Saurel-Cubizolles, M. Schweitzer, O. Thiebaugeorges.

**ELFE**

The authors are grateful to 1) the former members of the Elfe unit without whom the project would never have started: Henri Léridon, initiator and former Principal Investigator of the project, Stéphanie Vandentorren, Claudine Pirus, and Ando Rakotonirina; 2) the expertise and assistance of members of the unit for support functions, 3) all the researchers who contribute to the projects as members of the Elfe thematic groups and especially their coordinators; 4) all the field research assistants and interviewers; 5) and above all, all the Elfe families who have placed their confidence in us and given up their time to the study

**G21**

The authors gratefully acknowledge the families enrolled in Generation XXI for their kindness, all members of the research team for their enthusiasm and perseverance, and the participating hospitals and their staff for their help and support.

**GECKO**

The authors are grateful to the families who took part in the GECKO Drenthe study, the midwives, gyneacologists, nurses, and the general practitioners and all health professionals at the Preventive Child Healthcare Drenthe for their help in the recruitment and the measurements, and the GECKO Drenthe study team.

**Generation R**

The authors gratefully acknowledge the contribution of participants, research collaborators, general practitioners, hospitals, midwives, and pharmacies in Rotterdam.

**INMA**

The authors would particularly like to thank all the participants for their generous collaboration. The authors are grateful to Silvia Fochs, Nuria Pey, Mireia Garcia, Maria Victoria Estraña, Maria Victoria Iturriaga, Cristina Capo and Josep LLuch for their assistance in contacting the families and administering the questionnaires.

**MoBa**

The authors are grateful to all the participating families in Norway who take part in this on-going cohort study.

**NFBC1986**

The authors thank all cohort members and researchers who have participated in the NFBC studies. We also wish acknowledge the work of the NFBC project center.

**NINFEA**

The authors thank all families participating in the NINFEA cohort.

**The Raine study**

The authors would like to acknowledge the Raine study participants and their families for their ongoing participation in the study and the Raine study team for study co-ordination and data collection. We also thank the NHMRC for their long term contribution to funding the study over the last 30 years. The core management of the Raine study is funded by The University of Western Australia, Curtin University, Telethon Kids Institute, Women and Infants Research Foundation, Edith Cowan University, Murdoch University, The University of Notre Dame Australia and the Raine Medical Research Foundation.

**SWS**

The authors are grateful to the women and their children in Southampton who gave their time to take part in the Southampton Women’s Survey and to the research nurses and other staff who collected and processed the data.
