# Supplementary material for: Gestational age at birth and body size from infancy through adolescence: An individual participant data meta-analysis on 253,810 singletons in 16 birth cohort studies
Source: PLoS Med. 2023 Jan 26;20(1):e1004036. doi: 10.1371/journal.pmed.1004036 (PMC9879424; doi:10.1371/journal.pmed.1004036)
Supplement: Attachment — Submitted filename: Response to Reviewers.docx [file pmed.1004036.s020.docx]

**EDITOR COMMENTS**

1. I agree to the authors statement that they should not adjust for birth weight, since this is on the causal pathway. But maybe they should comment on why they don't analyze whether the associations partly are explained by small-for gestational age at birth, given available evidence within the DOHaD paradigm on intrauterine growth restriction and later nutritional status and health?

The included studies all represent high-income settings. It would be valuable to get comments on the limitation that low-or middle-income settings with a rapid nutritional transition are not represented, and what potential consequences this may have for the findings.

**Response:** Thanks for addressing these relevant points.

First, we are pleased that we are on the same page regarding adjusting for birth weight. We acknowledge it is also interesting to explore the influence of small-for gestational age at birth, and size for gestational age in general, also for this research question. For this study, however, the focus was deliberately on gestational age, without exploring the role of mediating factors such as birth weight, puberty or breastfeeding. The joint and separate associations with gestational age and size for gestational age is certainly of interest, and we are addressing this research question in another paper, using data from the Danish National Birth Cohort, as it was too complex to investigate in the 16 cohorts.

Second, we agree that including data from LMIC is relevant, with the proportions of preterm births and obesity being higher than in HIC. Unfortunately, we had no data available for LMIC, and have now addressed this limitation in the discussion.

2. Please report your IPD MA according to the PRISMA guidelines provided at the EQUATOR site. <https://eur02.safelinks.protection.outlook.com/?url=http%3A%2F%2Fwww.equator-network.org%2Freporting-guidelines%2Fprisma%2F&amp;data=05%7C01%7Cjohan.vinther%40sund.ku.dk%7C8a91658e03ba4f8438d408dac265c218%7Ca3927f91cda14696af898c9f1ceffa91%7C0%7C0%7C638036039566932108%7CUnknown%7CTWFpbGZsb3d8eyJWIjoiMC4wLjAwMDAiLCJQIjoiV2luMzIiLCJBTiI6Ik1haWwiLCJXVCI6Mn0%3D%7C3000%7C%7C%7C&amp;sdata=KqJ3aqRE2vSQexUNBUTmhkya0Poy0BN98INWj8NYMSg%3D&amp;reserved=0>

Please provide the completed PRISMA checklist. When completing the checklist, please use section and paragraph numbers, rather than page numbers. Please add the following statement, or similar, to the Methods: "This study is reported as per the Preferred Reporting Items for Systematic Reviews and Meta-Analyses (PRISMA) guideline (S1 Checklist)."

**Response:** The PRISMA checklist is a very informative and relevant tool for reporting systematic reviews. This study, however, is not a systematic review nor a conventional meta-analysis of previously published studies. Our study includes individual data meta analysis of 16 cohort studies conducted using a uniform analysis plan, and investigates a new research question, which has not been published before, in any of the 16 cohort studies. In that respect, we believe our study does not fit the PRISMA-checklist.

*DATA AVAILABILITY STATEMENT*

3. Please revise your data availability statement. Referring to another published manuscript regarding data access will not suffice. PLOS Medicine requires that the de-identified data underlying the specific results in a published article be made available, without restrictions on access, in a public repository or as Supporting Information at the time of article publication, provided it is legal and ethical to do so.

If the data are not freely available, please describe briefly the ethical, legal, or contractual restriction that prevents you from sharing it. Please also include an appropriate contact (web or email address) for inquiries (again, this cannot be a study author).

**Response:** Thanks for this. We have revised the data availability statement accordingly, and included appropriate contact detail for each cohort.

***TITLE***

4. Please revise your title according to PLOS Medicine’s style. Your title must be nondeclarative and not a question. It should begin with main concept if possible. "Effect of" should be used only if causality can be inferred, i.e., for an RCT. Please place the study design ("A randomized controlled trial," "A retrospective study," "A modelling study," etc.) in the subtitle (ie, after a colon).

**Response:** We believe that the title of our submitted manuscript already concur with the PLOS Medicine’s style, as it is non-declarative, not a question, without the ‘effect of’, but starting with a main concept.

*ABSTRACT*

5. Please report your abstract according to PRISMA for abstracts, following the PLOS Medicine abstract structure (Background, Methods and Findings, Conclusions)

<https://eur02.safelinks.protection.outlook.com/?url=http%3A%2F%2Fwww.plosmedicine.org%2Farticle%2Finfo%3Adoi%2F10.1371%2Fjournal.pmed.1001419&amp;data=05%7C01%7Cjohan.vinther%40sund.ku.dk%7C8a91658e03ba4f8438d408dac265c218%7Ca3927f91cda14696af898c9f1ceffa91%7C0%7C0%7C638036039566932108%7CUnknown%7CTWFpbGZsb3d8eyJWIjoiMC4wLjAwMDAiLCJQIjoiV2luMzIiLCJBTiI6Ik1haWwiLCJXVCI6Mn0%3D%7C3000%7C%7C%7C&amp;sdata=lnTNdKKJ315oLeykT2WEg4BkS2eERiB47Kk7ztUep4I%3D&amp;reserved=0>

**Response:** Please, see reply #2

6. Please provide details of the process of selection for the 16 population cohort studies included in your study - as you have done in main methods section. Please provide data sources and eligibility criteria.

**Response:** This is now added to the abstract

7. Please provide dates between which the 16 included studies were published

**Response:** Please, see reply to #2

8. Please include the important dependent variables that are adjusted for in the analyses.

**Response:** This is now added to the abstract

9. Please quantify the main results, p-values as well as with 95% Cis

**Response:** We argue that the p-values do not provide any new information that cannot be derived from the confidence intervals alone, and that they may be misleading as discussed by others (<https://www.nature.com/articles/d41586-019-00857-9>; (Sterne JA, Davey Smith G. Sifting the evidence-what's wrong with significance tests? BMJ. 2001 Jan 27;322(7280):226-31. doi: 10.1136/bmj.322.7280.226. PMID: 11159626; PMCID: PMC1119478). However, as the editor and the journal style require so, we added also p-values.

10. Please define GA at first use

**Response:** Gestational age at birth (GA) is defined at first use in Background (Line 62)

11. Please define OR and 95% CI at first use

**Response:** Odds Ratio (OR) and 95% Confidence Interval (CI) is now defined at first use in Methods and Findings

12. In the last sentence of the Abstract Methods and Findings section, please describe the main limitation(s) of the study's methodology.

**Response:** Thanks for addressing this. It is not included.

13. Please emphasize what is new without overstating your conclusions

**Response:** We have modified our conclusions accordingly

*AUTHOR SUMMARY*

14. At this stage, we ask that you include a short, non-technical Author Summary of your research to make findings accessible to a wide audience that includes both scientists and non-scientists. The Author Summary should immediately follow the Abstract in your revised manuscript. This text is subject to editorial change and should be distinct from the scientific abstract. Please see our author guidelines for more information:

<https://eur02.safelinks.protection.outlook.com/?url=https%3A%2F%2Fjournals.plos.org%2Fplosmedicine%2Fs%2Frevising-your-manuscript%23loc-author-summary&amp;data=05%7C01%7Cjohan.vinther%40sund.ku.dk%7C8a91658e03ba4f8438d408dac265c218%7Ca3927f91cda14696af898c9f1ceffa91%7C0%7C0%7C638036039566932108%7CUnknown%7CTWFpbGZsb3d8eyJWIjoiMC4wLjAwMDAiLCJQIjoiV2luMzIiLCJBTiI6Ik1haWwiLCJXVCI6Mn0%3D%7C3000%7C%7C%7C&amp;sdata=sJkVc9%2F3fwvNxDLgspMQT8m%2FdZr%2Bejre%2B1er%2BBoSsb0%3D&amp;reserved=0>

**Response:** Thanks for requesting this important addition to the submission. It is now added to the manuscript after the Abstract.

*METHODS and RESULTS*

15. We ask the following of all authors who submit a SR/MA, some may not apply to your IPD MA. Please respond in full to the below points to help facilitate and maximize transparency of data reporting. In any instance(s) where you are unable to respond to the request, please clearly state the reason(s) why not:
    a. Please update your included studies to the present time
    b. Please evaluate study quality and risk of bias
    c. Please evaluate evidence of publication bias
    d. Please provide dates between which the 16 included studies were published (as for the abstract).

**Response:** Many thanks for highlighting this, but we thing that the four points above (a,b,c,d) may not apply to our manuscript. We have described the reasons in the reply #2). Our study examines the association between gestational age at birth and body size (BMI, overweight) using individual participant data from 16 cohort studies. Although the study uses meta-analytic approach to analyze the study-specific estimates it cannot be considered as a standard meta-analysis of previous studies that requires an updated search of published studies, dates of publication and risk of publication bias.

16. Please provide the actual numbers of events for the outcomes, not just summary statistics or ORs.

**Response:** This is now added to the tables.

17. Please present numerators and denominators for percentages

**Response:** This is now added to the tables.

18. Where relevant, where you report 95% CIs, please also report p-values, including in the tables of the main manuscript and supplementary files.When a p-value is given please also provide the statistical test used to determine it. Please specify the significance level used (eg, P<0.05, two-sided). Please do not report p=0.000; report as p < 0.001

**Response:** This is now added to the manuscript and supplementary files

19. How was “maternal ethnicity” defined and by whom? Why was ethnicity considered important in this study and what it is believed to represent?

**Response:** Maternal ethnic background was defined by each cohort, locally and individuals were either categorized as western, non-western or mixed. The three categories were defined by the LifeCycle and the EU Child Cohort Network Harmonization protocol (please see de Moira et al 2021). More detailed information about how each cohort defined the variable is freely available in the Maelstrom Catalogue (<http://maelstrom-research.org>) for two cohorts (AOF, CHILD), and in the EU Child Cohort Network catalogue (<https://data-catalogue.molgeniscloud.org/catalogue/catalogue/#/>) for all other cohorts. Also, please see S1 Text for specific details in supplementary files. We have now referred to this in the paper.

We considered maternal ethnic background as a potential confounding factor, due to variations in gestational age between ethnic groups (https://pubmed.ncbi.nlm.nih.gov/23061689/), and variations in BMI and overweight between ethnic groups (https://www.bmj.com/content/372/bmj.n365).

20. Please update your ethics statement and include details of individual study ethical approval as a paragraph and not a list, in the main manuscript and not in the supplementary files. Please include in each case whether consent was written or oral.

**Response:** Our ethical statement is now outlined in the manuscript, and deleted from the supplementary files.

*FIGURES & TABLES*

21. Where you present adjusted analyses, please also present unadjusted analyses including in the supplementary figures.

**Response:** We have now included crude analyses in the main figures and supplementary figures.

22. Please also include p-values where you report 95% Cis, including supplementary figures

**Response:** This is now added to the manuscript and supplementary files

23. Please indicate, in each figure captions what the dot and whiskers represent in the forest plots

**Response:** This is now added to the manuscript and supplementary files

24. Please ensure that all abbreviations e.g. BMI and CI are defined within each figure caption in the main manuscript figures and supplementary figures.

**Response:** This is now added to the manuscript figures and supplementary files

25. Table 3: in the figure caption, please define BMI.

Table S4: please define β and β (se) in the caption

Please ensure that any colours you use in the figures are accessible to those with colour blindness (i.e. avoid the use of green and/or red)

**Response:** BMI is now defined in Table 3, and β and β (se) now defined in Table S4. Colours are now changed accordingly to avoid the use of green and red.

*DISCUSSION*

26. Please remove sub-headings from the discussion, including that which depicts the conclusions, such that the discussion reads as one continuous piece of prose.

**Response:** This is now removed.

27. Please remove the funding statement and conflict of interest statement form the end of the main manuscript and include only in the submission form.

**Response:** This is now removed

*REFERENCES*

28. Please see our website for reference guidelines here:

<https://eur02.safelinks.protection.outlook.com/?url=https%3A%2F%2Fjournals.plos.org%2Fplosmedicine%2Fs%2Fsubmission-guidelines%23loc-references&amp;data=05%7C01%7Cjohan.vinther%40sund.ku.dk%7C8a91658e03ba4f8438d408dac265c218%7Ca3927f91cda14696af898c9f1ceffa91%7C0%7C0%7C638036039566932108%7CUnknown%7CTWFpbGZsb3d8eyJWIjoiMC4wLjAwMDAiLCJQIjoiV2luMzIiLCJBTiI6Ik1haWwiLCJXVCI6Mn0%3D%7C3000%7C%7C%7C&amp;sdata=FUzHzfV%2FLw4C6fLcyMfRiP7%2Bn4FZI1k%2FrMVV8TYmBDU%3D&amp;reserved=0>

Citations should be placed within square parentheses preceding punctuation. Where more than one article is cited please separate the citations with a comma and no spaces as follows: [1,2,3,4] or [1-3,5,7] Journal name abbreviations should be those found in the National Center for Biotechnology Information (NCBI) databases. In the reference list please ensure no more than 6 author names are listed followed by et al. where more than 6 co-authors contribute to the study.

**Response:** The references in the manuscript are now updated accordingly

**REVIEWER COMMENTS**

**REVIEWER #1:**

The authors investigated the association of GA with BMI z-score and odd of overweight from infancy through adolescence. They found interesting results that higher GA is important for higher BMI in infancy, while the association attenuate with age and preterm children have a similar mean BMI to those born term.

Major points

29. I could not understand how you calculated BMI z-score during infancy as in references (68) and (69) because there was no BMI z-score data for under five years.

**Response**: Many thanks for addressing this point, which was inadequately addressed in the revised version. We used the cut-offs from WHO (reference #68 for <5 years, and reference #69 for ≥5 years) and calculated the BMI z-scores in infancy, childhood and adolescence using the instructions and guidelines from *Vidmar SI, Cole TJ, Pan H* (2013) *“Standardizing anthropometric measures in children and adolescents with functions for egen: Update”. The Stata Journal, 13:2, 366-378* (<https://journals.sagepub.com/doi/10.1177/1536867X1301300211>). Please, see Table 5, p. 374, regarding BMI (0-19 years). The manuscript is now updated accordingly, and the reference is now added (reference #68) to the manuscript.

30. I think you should add the data of small for gestational age at birth and analyze the influence of SGA.

**Response:** Thanks for addressing this, which is indeed a limitation in the study. Please see reply #1.

**REVIEWER #2:**

This is a well-conducted study on the association between gestational age at birth and body size from infancy to adolescence in 16 birth cohort studies. The study design, datasets, statistical methods and analyses, and presentation (tables and figures) and interpretation of the results are mostly adequate. However, there are still a few major issues needing attention.

31. Year of birth. From Table 1 we can see the year of birth ranges from 1985 to 2017 - across 33 years for the 16 cohort studies. This big time difference needs to be addressed in the analyses as the landscape of perinatal mortality, morbidity and healthcare has changed a lot over time. However, year of birth was not adjusted at all in any of the analyses in the paper which is inadequate. Firstly, year of birth need be adjusted in all the analyses; secondly, analyses such as meta regression on whether this is a time trend of the results are needed.

**Response:** We thank the reviewer for addressing this point. We agree that it is important to assess whether year of birth, which ranges over three decades in our study, could have an impact on the main findings. We have now examined the influence of this – by adjusting the analyses for birth year, and the main findings remained unchanged. We further performed stratified analyses by year of birth – diving children before/after 2001, which is now presented in supplementary files. The stratified analyses showed no difference in the estimates for almost any of the outcomes. The only exception was GA and overweight at >14-19 years, where estimates for children before 2001 (OR: 1.00 [95% CI: 0.98, 1.02]) slightly differed from those born in or after 2001 (OR 0.94 [95% CI: 0.91, 0.98]). However, only one cohort contributed to data after 2001 (DNBC, Denmark) in this age-band, and with relatively small sample size. Overall there was no evidence of an interaction with birth year, and this (and the above) is now addressed in the discussion part.

32. Interpretation of results for adolescence. One of the key conclusions in the abstract is "By adolescence, preterm children have on average a similar mean BMI to those born term". However, as shown in figure 2 (also figure 1), there are only 4 studies included in the analysis for adolescent outcomes as compared to 16 or so studies for analyses of earlier years' outcomes, therefore the sample size is substantially reduced for the analyses for this adolescent category, which may impact on the statistical significance of the results. This issue happens to all the analyses for the adolescence in the paper. For example, in figure 2, the no difference in mean BMI between preterm and term for adolescence may be due to the very wide 95% CIs caused by smaller sample size so that the results might be inconclusive due to uncertainty. Overall, this is a limitation of the study and the results need to be read with caution.

**Response:** Thanks for emphasizing this. We have changed the text accordingly in the abstract, where we acknowledge that the sample size at age >14-19 years is reduced as to date only children from few included cohorts reached the adolescence. Although the sample size is reduced compared with analyses on younger age groups, it is still relatively large compared with previous studies on GA and BMI in adolescence. Also, our findings are in line with majority of existing evidence reporting similar BMI between preterm and term, around the same age.

33. Missing data. The missing percentages are shown in S3 Table and we can see some studies have high missing rates in some variables. It looks like the authors have used available data for analyses. Have the authors dealt with the missing data in any way? If not, what are the impact and limitation to the results caused by missing data potentially?

**Response*:*** We acknowledge that missing data is an issue in the current study. At this stage, however, imputation or alternative methods to deal with missing data is under development and is currently not possible in DataSHIELD. There was only few missing data on our exposure (GA), which is positive. For the outcomes (BMI, overweight), there is issues with missing as not measured or reported, and with attrition at different time points. As shown in S3 Table, there is issue with missing data (exceeding 10%) on covariates in few cohorts (e.g. maternal education, pre-pregnancy BMI, smoking in pregnancy, gestational hypertension), which may bias our findings. We have explicitly addressed this as a limitation in the discussion.

34. 'Leave one out' analysis is a good option for sensitivity analysis. The results are shown in S2 figure but a bit difficult to follow. For example, in S2 figure A, what's estimate for each cohort study? Does it mean leave that named study out? Basically, I'd like to see the results without the BIB study from the UK as it shows very different characteristics and outcomes (and also missing data) from the others as shown in table 1 and S3.

**Response:** Thanks for addressing this point. We performed ‘leave-one-out’-analyses for both BMI and Overweight. It is correct that the estimates illustrated represent the analysis without that specific cohort, i.e. for S2 A, leaving out BIB showed similar findings (0.01 without, 0.02 with). In the right side of the figures, weight (%) are presented. For BiB, the weights are generally small, hence may explain why excluding BiB has no marked impact.

**REVIEWER #3:**

This is an important study using data from multiple cohorts addressing the association between gestational age and later BMI. The paper is well-written, and the methodology is fine.

I have several comments:

35. The ethical paragraph is too brief. In fact, the authors only refer to the supplements for a description about the MREC/IRB approval and the consent procedure of each study. This is insufficient. The ethical paragraph should describe the grounds for allowance of data sharing with an international consortium. More specifically, did parents give written informed consent for data sharing, or was a waiver of consent for data sharing provided (by the local MREC/IRB or the local privacy officer) under the assumption that data could impossibly identify individual subjects (eg, due to the large sample size, sharing of minimal data)? In this regard, it is also important to clarify who was the receiving party.

**Response:** We agree, and we have extended the ethical paragraph adding more thorough information. The current study, and generally the studies conducted within the EU Child Cohort Network use the federated analyses that involve no data sharing, but only meta-data is made accessible upon request. Also, each cohort included in the current study obtained parental informed consent for using the data. This is now explained thoroughly, and in detail in the revised manuscript.

36. I would be more careful with the conclusion that "by adolescence, preterm children have on average a similar mean BMI to those born full term". There is no follow-up beyond 19 years, implicating that the observed catch-up in BMI after prematurity, as observed at age categories 9-14 and 14-19, might be determined by earlier pubertal timing. There is some evidence suggesting that children born preterm have an earlier onset of pubertal growth velocity (Wehkalampi, et all. JCEM 2011), although Tanner pubertal stages and menarcheal age are generally age-appropriate (Ford, et al. Arch Pediatr Adolesc Med 2000; Peralta-Carcelen, et al. J Pediatr 2001; Hack, et al. Pediatrics 2003; Saigal, et al. Pediatr Res 2006). The authors should add to the Discussion that pubertal timing might play a role in the associations observed.

**Response:** Thanks for pointing this out. Pubertal onset is a potential mediator of the association between GA and BMI in adolescence, and as such should not be adjusted for in the analyses. We acknowledge, however, that it could be interesting to further explore, and that we did not do so. This is mentioned in the revised manuscript – in line 198-199 in method section.

37. Limitations are generally well acknowledged, but in view of my previous comment some issues should be added to this section, including (1) the lack of follow-up extending into adulthood, (2) the low proportion of preterm born children, and (3) the lack of information on puberty status (at least for the age categories 9-14 and 14-19).

**Response:** Thanks for this. Please, see reply #36 for (1) and (3). As regard (2) – in a global context, Europe has the lowest proportion of preterm births, hence the results are not surprising (*Chawanpaiboon S, Vogel JP, Moller AB, Lumbiganon P, Petzold M, Hogan D, et al. Global, regional, and national estimates of levels of preterm birth in 2014: a systematic review and modelling analysis. Lancet Glob Health. 2019;7(1):e37-e46*). However, in one cohort, individuals were recruited in the 3^rd^ trimester, which may lead to exclusion of some individuals born preterm. We have addressed this limitation in the last part of the discussion.

38. I am pleased to read that the authors clearly state in the Abstract and in the Introduction that birth weight cannot be used as a proxy for gestational age, a mistake that has been made by many previous studies, eg, due to lack of accurate gestational age determination in older cohorts. It would be nice if the authors could place their findings into (historical) context in the Discussion.

**Response***:* Many thanks for agreeing on this point, together with the editor and the other reviewers. We agree it is important to place this in a broader context, and have now addressed this explicitly in the discussion.

**REVIEWER #4:**

Summary and Recommendation:

1) This study provides a novel approach to analyzing a unique dataset from the EU Child Cohort Network to investigate the relationship of gestational age/prematurity to later body size from infancy through adolescence. By including individual data on 253,810 children from 16 cohorts across a wide range of gestational ages and follow-up ages and by controlling for covariates and assessing heterogeneity, this study aims to overcome a number of methodological limitations of prior published studies, reviews and meta-analyses. The result is a robust evaluation of a large and diverse population, though it is still limited by a number of factors, including those which the authors point out as well as a number of other issues that need to be further addressed.

2) The authors employ a linear regression model to exam the associations of GA in weeks and in clinical categories (5 groups ranging from very preterm to post-term) with BMI z-scores across 6 age bands (ranging from early infancy to adolescence); and they employ a binomial logistic regression model to examine the relationship of GA and of clinical categories to overweight (defined as >2 SD above WHO Child Growth Standard median for children < 5yo and >1 SD above WHO Growth Reference median for children >=5yo) across similar age bands. Both sets of analyses control for multiple a priori selected factors that were known or plausible causes of variation in GA and subsequent body size, with final selection based on a directed acyclic graph. For 8 cohorts where multiple growth measurements were available for the same child in one or more of the six age-groups the latest measurement was chosen, presumably in order to treat last age of measurement as a cross-sectional variable across all cohorts.

3) There were multiple differences in cohort-specific sample size, distributions of covariates, gestational age groups and follow-up age groups. The proportion of children classified as overweight also varied between cohorts in each age group.

4) Given this heterogeneity, the authors performed meta-regression analyses to determine which study characteristics were independently associated with between-study (cohort) heterogeneity and also performed a 'leave-one-out' analysis for cross-validation.

5) Would recommend revisions to address issues noted below

Evidence and Examples

Major Issues:

39. In addition to the heterogeneity that was analyzed, there was also variation in sources of growth data as delineated in Table S1, including at least 4 studies where the source was parent report, including the two largest cohorts from Denmark and Norway (which together comprise over 2/3 of the total sample). A further analysis to compare data from the studies with actual measurements with those from studies with parent-reported measurements would be important.

**Response*:*** Thanks for addressing this. We agree it is important, and especially in this study, to analyze the role of heterogeneity. We have already at this stage made quite a few analyses to examine and explore heterogeneity, and agree that it could also be interesting to explore additional ones, including the role of method used to measure growth. We do acknowledge that this may add information to our findings, but at this stage we would need to request all 16 cohorts new data on this. Hence, we added this accordingly as a limitation to the discussion, while supported by a reference to a systematic review <https://onlinelibrary.wiley.com/doi/epdf/10.1111/j.1467-789X.2007.00347.x>.

40. There was also heterogeneity in years of birth (ranging from 1985 to 2017 and presumably also in years at last follow-up measurement. As the authors suggest in the discussion this may reflect some changes over time in population and newborn and subsequent management; but no analysis by year of birth is provided.

**Response*:*** Thanks for addressing this relevant and interesting point. Please see reply #31 for our response.

41. The percent of children born preterm ranged from 3.1% to 7.5%, which is relatively low compared with other reports (even excluding extremely preterm infants), with 4 cohorts including <1% very preterm.

**Response*:*** Thanks for also addressing this . Please see reply #37 for our response.

42. Granted following children up through adolescence is challenging, the body size data for >14 - 19 yo was based on only 4 studies, with over ¾ of those coming from one cohort (Denmark). This needs to be mentioned in limitations section.

**Response*:*** Thanks for raising this point. Please see reply #32 for our response.

43. The results for BMI scores indicate that increasing GA is associated with increasing BMI only during later infancy/early childhood, with progressive attenuation up through adolescence. Conversely, the more premature the clinical categories the lower the BMIz compared to term (for very preterm this was observed at all ages up until adolescence and for late until pre-adolescence). At first glance this appears paradoxical, but on further consideration it is likely related to the different "directions" of the comparison (higher GA compared with lower in the first case and lower compared with higher (term) in the second. That said, this would be worth clarifying further in the discussion.

**Response*:*** Thanks for highlighting this. This is now clarified in the discussion

44. A similar relationship of GA to overweight was found, with attenuation through childhood to adolescence; but in contrast to BMI results and to overweight results by GA, very preterm infants had an increased risk of overweight in adolescence. Though the latter is consistent with several other cited studies from Finland and Australia, the reason for this shift at adolescence is not clear from the data presented and though the discussion points this out, it does not elucidate further. Several recent studies of extremely preterm infants found high prevalence of obesity at pre-adolescence or adolescence, but no difference in prevalence compared to term controls (a study from EPICURE network in England Yanyan Ni et al Arch Dis Child Fetal Neonatal Ed. 2020 Sep;105(5):496-503 and a study from Dallas Jessica Wickland et al Pediatr Res April 2022); yet other co-morbidities were found elevated. This would be worth a brief comment.

**Response*:*** We agree that much research, especially in recent years, focus on the health outcomes of preterm individuals, including cardio-vascular risk factors (e.g. height, weight, BMI and overweight) and other co-morbidities. Yanyan Ni et al is one of them, and mistakenly not mentioned in the discussion of manuscript. It is now explicitly addressed in both the background and discussion. Also, we acknowledge that several studies have reported on overweight in preterm infants, yet studies are generally heterogeneous in classifying and defining the infants born preterm, which is also highlighted in the manuscript (Lurbe E, Ingelfinger J. Developmental and Early Life Origins of Cardiometabolic Risk Factors: Novel Findings and Implications. Hypertension. 2021;77(2):308-18).

Minor Issues:

45. Results note several associations in linear regression analyses whose confidence intervals include 0; strictly speaking such associations are not statistically significant, even though the estimated mean difference in BMIz is >0.0. Moreover, the Forest Plots for these results in Figure 1 do not clearly show inclusion of x axis =0 in the plots.

**Response*:*** The axis is now more clearly shown.

46. Likewise, in the results from the logistic regression, several associations are noted which include 1 in the confidence interval; strictly speaking such associations are not statistically significant, even though the odds ratio is>1.0.

**Response*:*** While we, indeed agree that some of the results are statistically non-significant, and we always show the corresponding confidence interval to make this clear. However, we argue that the overall findings should not be interpreted by looking at the arbitrarily imposed p-value cut-off of 0.05, but should rather be seen in the light of their robustness and the consistency of the estimates (Sterne JA, Davey Smith G. Sifting the evidence-what's wrong with significance tests? BMJ. 2001 Jan 27;322(7280):226-31. doi: 10.1136/bmj.322.7280.226. PMID: 11159626; PMCID: PMC1119478).
