# Supplementary material for: Gestational age at birth and body size from infancy through adolescence: An individual participant data meta-analysis on 253,810 singletons in 16 birth cohort studies
Source: PLoS Med. 2023 Jan 26;20(1):e1004036. doi: 10.1371/journal.pmed.1004036 (PMC9879424; doi:10.1371/journal.pmed.1004036)
Supplement: Attachment — Submitted filename: TO EDITOR_14_12_2022.docx [file pmed.1004036.s021.docx]

**RESPONSE TO EDITOR**

GENERAL

Thank you for your considered and detailed response to previous editor and reviewer requests, all of which are satisfactory upon review.

- Many thanks, we appreciate your feedback

The editorial team notes that upon comparison of your manuscript versions many, of the required changes, despite being detailed clearly in your responses were not evident in the manuscript – for example, the author summary is still absent, some figures appear unchanged, changes to the abstract are not apparent.

- We sincerely apologize for this. As specifically outline to the points below, we have again looked at the revised manuscript and figures (dated 30NOV22), and actually this includes an author summary, updated references, and the figures also include p-values and unadjusted estimates.
- Please, let us know if something is not apparent from your side, once you receive this new revised manuscript.

*** Please see below for specific examples and please check carefully against ALL previous requests to ensure that all changes have been included in the manuscript ***

TITLE

We note your response to the editorial request to update your title. Please revise your title such that it complies with our journal requirements. It should be defined as an individual participant data meta-analysis. Suggest the following:

“Gestational age at birth and body size from infancy through adolescence: an individual participant data meta-analysis of 253,810 singletons from 16 birth cohort studies”

- The title is changed accordingly, and now comply with the journal requirements. Thank you.

ABSTRACT

We requested that p-values be added to the data reporting, which is a journal requirement, as you acknowledged in your response. We could not see any p-values in the abstract. Please add the p-values. Please also ensure that CIs are reported consistently, currently upper and lower limits are separated either by a semi-colon or a colon and should be separated by a comma (or hyphen although this can cause confusion with negative value reporting) please revise accordingly and throughout the main manuscript.

- We apologize. P-values are now added to the abstract, and the CIs are reported consistently. This is also revised throughout the main manuscript

We requested that the main limitations of the study’s methodology be detailed at the end of the methods and findings, but we could see it. Please include this in the abstract.

- This is now added to the abstract, in the last sentence before conclusion.

AUTHOR SUMMARY

We could not see an author summary in the main manuscript, please include as previously requested following the abstract

- We just checked the version online, and this was actually included in the previous revised manuscript.. Hence, it is also included in this revised manuscript (dated 14 December 2022)

METHODS and RESULTS

Many of the figures are unchanged compared to previous versions – for example unadjusted analyses are not present, p-values are absent from the main manuscript

- As addressed above, the figures were updated last time, and included. We added unadjusted analyses and p-values. Also p-values are added throughout the main manuscript now.

REFERENCES

We previously asked:

“Please see our website for reference guidelines here: <https://eur02.safelinks.protection.outlook.com/?url=https%3A%2F%2Fjournals.plos.org%2Fplosmedicine%2Fs%2Fsubmission-guidelines%23loc-references&amp;data=05%7C01%7Cjohan.vinther%40sund.ku.dk%7Cdc8734b796d9459e4cfc08dadc70769a%7Ca3927f91cda14696af898c9f1ceffa91%7C0%7C0%7C638064672826588944%7CUnknown%7CTWFpbGZsb3d8eyJWIjoiMC4wLjAwMDAiLCJQIjoiV2luMzIiLCJBTiI6Ik1haWwiLCJXVCI6Mn0%3D%7C3000%7C%7C%7C&amp;sdata=3E8tBVwUDIKGZKgSqtZK0O9OusTZnzUQlV4c85%2BBpQE%3D&amp;reserved=0>

Citations should be placed within square parentheses preceding punctuation. Where more than one article is cited please separate the citations with a comma and no spaces as follows: [1,2,3,4] or [1-3,5,7] Journal name abbreviations should be those found in the National Center for Biotechnology Information (NCBI) databases.

In the reference list please ensure no more than 6 author names are listed followed by et al. where more than 6 co-authors contribute to the study.” These changes were not evident, please amend as previously requested

- In the revised manuscript (30NOV22), references were updated. Please, find it also it in this manuscript.
